# Association of serum AFP trajectories and hepatocellular carcinoma outcomes after hepatic arterial infusion chemotherapy: A longitudinal, multicenter study

**DOI:** 10.1002/cam4.7319

**Published:** 2024-05-31

**Authors:** Chao An, Ran Wei, Wang Yao, Wenwen Han, Wang Li, Ge Shi, Peihong Wu

**Affiliations:** ^1^ Department of Minimal Invasive Intervention State Key Laboratory of Oncology in South China, Collaborative Innovation Center for Cancer Medicine, Sun Yat‐Sen University Cancer Center Guangzhou China; ^2^ Department of Gastrointestinal Surgery The First Affiliated Hospital of Sun Yat‐Sen University Guangzhou China; ^3^ Department of Interventional Oncology The First Affiliated Hospital of Sun Yat‐Sen University Guangzhou Guangdong China; ^4^ Department of International Radiology and Vascular Surgery The First Affiliated Hospital of Jinan University Guangzhou China; ^5^ Medical Cosmetic and Plastic Surgery Center, The Sixth Affiliated Hospital, Sun Yat‐Sen University Guangzhou China

**Keywords:** AFP trajectories, hepatic arterial infusion chemotherapy, hepatocellular carcinoma, survival outcomes, the latent class growth mixed model

## Abstract

**Aim:**

This study aims to investigate α‐fetoprotein (AFP) trajectories for prediction of survival outcomes after hepatic arterial infusion chemotherapy (HAIC) treatment in large hepatocellular carcinoma (HCC).

**Methods:**

From May 2014 to June 2020, 889 eligible patients with large HCC underwent HAIC were retrospectively enrolled from five hospitals. A latent class growth mixed (LCGM) model was applied to distinguish potential AFP level dynamic changing trajectories. Inverse‐probability‐of‐treatment weighted (IPTW) analyses were performed to eliminate unmeasured confounders through marginal structural models. Multivariate Cox proportional hazard regression analyses were used to determine the overall survival (OS) in patients with large HCC. Performance of these serum markers for survival prediction was compared by areas under receiver operating characteristic analysis with the Delong test.

**Results:**

The median follow‐up time was 23.7 (interquartile range, 3.8–115.3). A total of 1009 patients with large HCC, who underwent HAIC with AFP repeatedly measured 3–10 times, were enrolled in the study. Three distinct trajectories of these serum AFP were identified using the LCGM model: high stable (37.0%; *n* = 373), low stable (15.7%; *n* = 159), and sharp‐falling (47.3%; *n* = 477). Multivariate Cox proportional hazard regression analyses found that ALBI stage 2–3, BCLC‐C stage and high‐stable AFP trajectories were associated with OS. AFP trajectories yield the optimal predictive performance in all risk factors.

**Conclusions:**

The AFP trajectories based on longitudinal AFP change showed outstanding performance for predicting survival outcomes after HAIC treatment in large HCC, which provide a potential monitoring tool for improving clinical decision‐making.

## 1 INTRODUCTION

Large hepatocellular carcinoma (HCC) (diameter >5 cm) remains a great challenge to curative surgical resection (SR).^1^ Even if the R0 margin of SR is performed completely, the postoperative early recurrence rate remains extremely high, exceeding 30%.^2^, ^3^ With the increase in tumor diameter, the possibility of tumor thrombus formation also increases, according to previous reports.^4^ Furthermore, targeted chemotherapy and immune checkpoint inhibitor therapy also have difficulty reversing the poor survival outcomes of these patients.^5^, ^6^, ^7^ Although transarterial chemoembolization (TACE) is the current standard of care therapy for patients with intermediate advanced‐stage HCC, patients with large HCC derive considerably less benefit.^8^ Once the tumor burden reaches beyond the up‐to‐seven criteria, the phenomenon of TACE resistance will also likely occur.^9^ Recently, hepatic arterial infusion chemotherapy (HAIC), as a promising option that provides sustained local high concentrations of the chemotherapy agents into the tumor, outperforming intravenous administration, has received extensive support in the effective and safe treatment of intermediate advanced‐stage HCC.^10^, ^11^, ^12^, ^13^ In Japan, HAIC has been recommended as the first‐line treatment for HCC with portal vein tumor thrombosis (PVTT). Notably, Shi Ming et al. showed that HAIC with the FOLFOX regimen yielded a higher median OS (23.1 months) and ORR (48%) than TACE treatment in large HCC (largest diameter >7 cm) patients in a randomized phase III trial.^14^

However, large HCC comprises a wide range of heterogeneous populations.^15^ Even if HAIC treatment has several advantages, including the operation being standardized, the chemotherapy scheme being unified (FOLFOX, oxaliplatin plus fluorouracil, and leucovorin), and the sessions being regular (a 21‐day interval), it may result in different survival benefits among different HCC populations. In particular, the incidence rate of tumor progression after HAIC varies widely, which also brings great challenges to the decision‐making and adjustment of sequential treatment schemes. α‐Fetoprotein (AFP), as the most common biomarker used for detecting HCC, is a trustworthy oncofetal biomarker. The AFP level can also provide hints for the therapeutic effect in clinical practice and has been used to predict the risk of tumor recurrence after SR, liver transplantation, locoregional therapy, and systemic therapy.^16^, ^17^ To further explore the new utility of this conventional biomarker, the AFP response (>20% decrease after initial therapy) was employed to predict the radiologic response and survival of HCC patients undergoing targeted chemotherapy.^18^, ^19^ However, owing to the complex and changeable AFP trend in patients with large HCC who underwent multiple‐cycle HAIC treatment, the conventional cutoff value used to distinguish high and low levels of AFP also has difficulty accurately predicting the final survival outcome, especially postoperative tumor progression.

In view of the principle of multiple‐cycle, regular and standardized HAIC treatment, the prediction of survival outcome by AFP level at different time points and trajectories remains controversial. Recently, some longitudinal landmark models have been commonly used to predict oncological outcomes by longitudinal biomarker data, obtaining predictions at any postbaseline time. For example, Yayuan Zhu et al. developed a dynamic risk assessment model for predicting the risk of kidney failure based on multiple longitudinal biomarker processes.^20^ Linbin Lu et al. used a predictive model based on the trajectories of serum AFP levels to evaluate the survival outcomes of HCC patients who underwent TACE.^21^ Therefore, we aimed to investigate the predictive ability of AFP trajectories for HAIC treatment in large HCC in a longitudinal, multicenter study. Furthermore, we aimed to confirm whether AFP trajectories are applicable to the prognosis prediction of HAIC treatment for large HCC with different BCLC stages.

## 2 METHODS

We present the following article following the Strengthening the Reporting of Observational Studies in Epidemiology (STROBE) Statement. This retrospective, multicenter study protocol was approved by the Institutional Review Board of all participating hospitals and conducted in accordance with the principles of the 1975 Helsinki Declaration. The requirement for informed consent per patient was waived due to the retrospective nature of the study.

### 2.1 Study design

**FIGURE 1:**
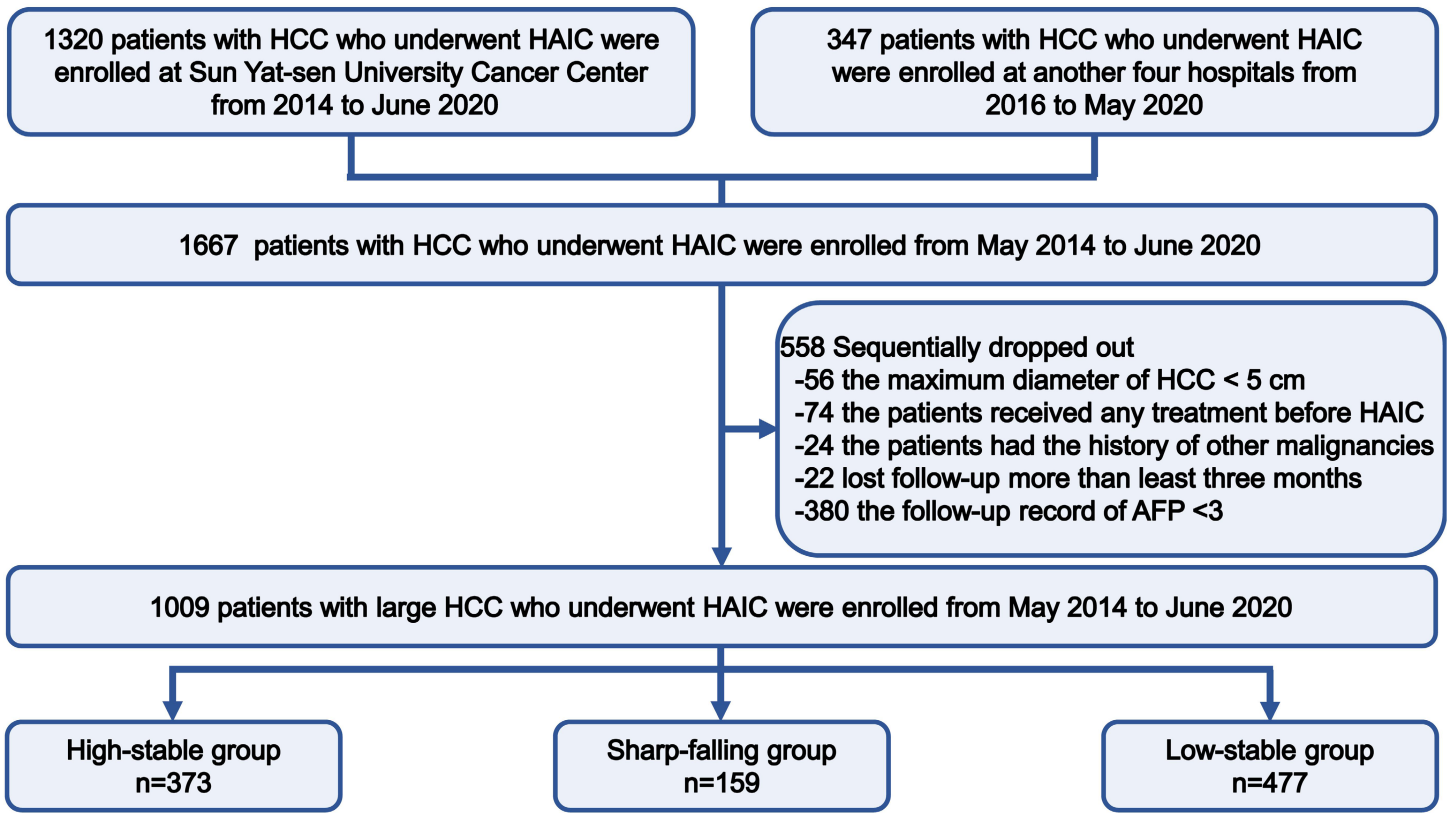
Flowchart for patients with large hepatocellular carcinoma after HAIC. AFP, α‐fetoprotein; HAIC, hepatic arterial infusion chemotherapy; HCC, hepatocellular carcinoma.

Between February 2014 and April 2022, a total of 1667 HCC patients with pathologically confirmed disease who subsequently initially underwent HAIC were enrolled at five hospitals. HCC was diagnosed based on the guidelines of the American Association for the Study of Liver Diseases (AASLD) and the European Association for the Study of the Liver (EASL).^22^, ^23^ Furthermore, some suspected cases were confirmed by a needle biopsy. HAIC was recommended as an alternative treatment to TKIs in advanced HCC based on previous phase II and phase III studies. According to the patient recruitment pathway (Figure 1), the inclusion criteria were as follows: (i) age 18–75 years old; (ii) Eastern Cooperative Oncology Group (ECOG) performance score <2; (iii) Child‐Turcotte‐Pugh (CTP) grade A and B; and (iv) adequate blood or bone marrow (leukopenia count >3.0 × 10^9^/L, platelet count >60 × 10^9^/L, and hemoglobin >8.0 g/L). The exclusion criteria were as follows: (i) history of other malignancies; (ii) combination with other treatments before HAIC; (iii) lost to follow‐up >3 months after HAIC; and (iv) clinical data missing AFP trajectories. The HAIC procedure, modified FOLFOX6 regimen, and criteria for protocol treatment discontinuation are described in detail in Appendix S1–S3.

### 2.2 Follow‐up protocol and AFP measurement

All patients were censored at the last follow‐up date (October 30, 2022). If a thorough HAIC procedure was accomplished, then serum AFP was examined again at 1 week after HAIC and at approximately 2‐ to 4‐week intervals during the whole follow‐up period in the first 1 year. After 1 year of remission, the frequency gradually decreased to every 3 months. The serum AFP level was measured by electrochemiluminescence immunoassay using the Roche Cobas E602 system (Roche Diagnostics GmbH, Mannheim, Germany) according to the manufacturer's instructions. The cutoff value of AFP for HCC was set at 400 ng/mL. Preoperative serum AFP was defined as the AFP value closest to the first HAIC treatment within the first follow‐up record. Postoperative serum AFP included the AFP value before any treatment at each follow‐up record after the first HAIC. The results of repeat AFP tests per patient with a follow‐up record were excluded.

### 2.3 Outcome assessment and covariate selection

Patient death or disease progression was set as the end point of this study. The date from the initiation of HAIC to the date of the death or end of the follow‐up was applied for the calculation of the overall survival (OS). The date from the first HAIC to the date of tumor progression based on modified Response Evaluation Criteria in Solid Tumor (mRECIST) or the end of the follow‐up was applied for the calculation of progression‐free survival (PFS). The date from the first HAIC to the date of intrahepatic tumor recurrence (abnormal nodules, disseminated, and/or unusual patterns of peripheral enhancement in the liver parenchyma) or end of the follow‐up was applied for the calculation of intrahepatic recurrence‐free survival (IRFS). Twenty‐one covariates were selected to adjust for the associations between their trajectories and OS, PFS and IRFS, which are shown in Appendix S4.

### 2.4 Statistical analysis

Even though the AFP level was standardly evaluated across all cohorts, random interference could still affect the results. We employed a latent class growth mixed model (LCGMM) to divide HCC patients into many distinct groups, each of which has the same trajectory pattern of serum AFP levels at various times and may be distinguished by AFP trajectory trends. Logarithmic transformation was used for adjusting serum AFP levels because of its left skewness (AFP all referred to log (AFP) hereafter). The longitudinal measurements were set as linear or nonlinear functions of time (weeks between each measurement date and the surgery date). Various trajectory shapes were examined using three different polynomials, namely, linear, quadratic, and cubic polynomials, to account for both linear and nonlinear AFP patterns. Repeated trajectory analysis was carried out for each specification of trajectory shape by switching the class number ranging from 2 to 6 and using the identical beginning values derived from the 1‐class model. LCGMM models with 2–6 classes were run multiple times using various sets of random beginning values based on the 1‐class model to prevent convergence toward local maxima.^24^, ^25^ We determined the best‐fit model together with the study‐specific requirements based on the following criteria^24^, ^26^: (a) significant improvement in the Akaike information criterion (AIC) and Bayesian information criterion (BIC); (b) high mean posterior class membership probabilities (>0.70); (c) high posterior probabilities (>0.70); and (d) ≥5% membership in any single trajectory group. The cubic trajectories of the three groups were deemed to be the best match model in this investigation. The “lcmm” R package was used to establish the model in R 4.2.0.

Characteristics across several groups were compared using the Kruskal–Wallis test or Student's *t* test for continuous variables and *X* ^2^ statistics or Fisher's exact test for categorical variables, described as numbers (%). The OS, PFS, or IRFS estimated by the Kaplan–Meier method was used to establish the survival curve for each trajectory group, and the log‐rank test was performed to assess the overall differences between the groups. The significant risk factors were included in univariate and multivariate Cox proportional hazards regression models. The correlation matrix was used to assess the related variables for collinearity and identify plausible interaction terms, including interactions between different risk factors. The associations between trajectory groups and HCC outcomes were adjusted by multivariable Cox regression hazard models. Specifically, Fine‐Gray competing risk regression analysis was employed to assess the cumulative incidence of intrahepatic tumor recurrence. Experiencing extrahepatic metastasis and dying of all causes were considered competing events by calculating hazard ratios (HRs) and 95% confidence intervals (CIs) for intrahepatic tumor recurrence. We employed restricted cubic spline to address the nonlinearity of confounding factors and evaluated the relative importance of each parameter to OS, PFS, and IPFS using *Χ* ^2^ from the “rms” R package.

### 2.5 Sensitivity analysis

Through the use of marginal structural models, inverse‐probability‐of‐treatment weighted analysis (IPTW) was carried out to remove the unmeasured confounding factors. To evaluate potential heterogeneity and stabilized IPTW, we stratified the participants by age, ALBI stage, tumor size, tumor number, vascular invasion, BCLC stage, and extrahepatic metastasis, repeated the Cox or Fine‐Gray competing risk regression analysis in each subgroup, and included the multiplication term to test the existence of interaction. To account for potential biases of the various follow‐up times, sequential landmark analyses evaluating survival with distinct AFP trajectories were performed for patients with OS times of <1‐year, 2‐years, and 3‐years.

## 3 RESULTS

### 3.1 Patient cohorts

After exclusion, a total of 1009 treatment‐naïve HCC patients (110 females and 899 males; median age: 51.5, interquartile range: 42.0–59.0) were enrolled in the trajectory analysis of dynamic AFP. The median follow‐up time was 23.7 (interquartile range, 3.8–115.3) months in this study. The longitudinal measurement duration of the AFP trajectory fitting was reexamined at intervals of roughly one to 4 weeks after HAIC, which was divided into three types of AFP trajectories and is shown in Figure 1. The baseline characteristics of the HCC patients are shown in Table S1. Most patients' partial leading cause of HCC was HBV infection (92.5%). The numbers of BCLC A, B, and C stage HCC patients were 88 (8.7%), 140 (13.9%) and 781 (77.4%), respectively. Notably, most HCC patients who underwent HAIC were found to be in an advanced stage. Some examples of patients with large HCC receiving HAIC combination therapy are shown in Figure S1–S3.

### 3.2 Identification of the number of trajectories

**FIGURE 2:**
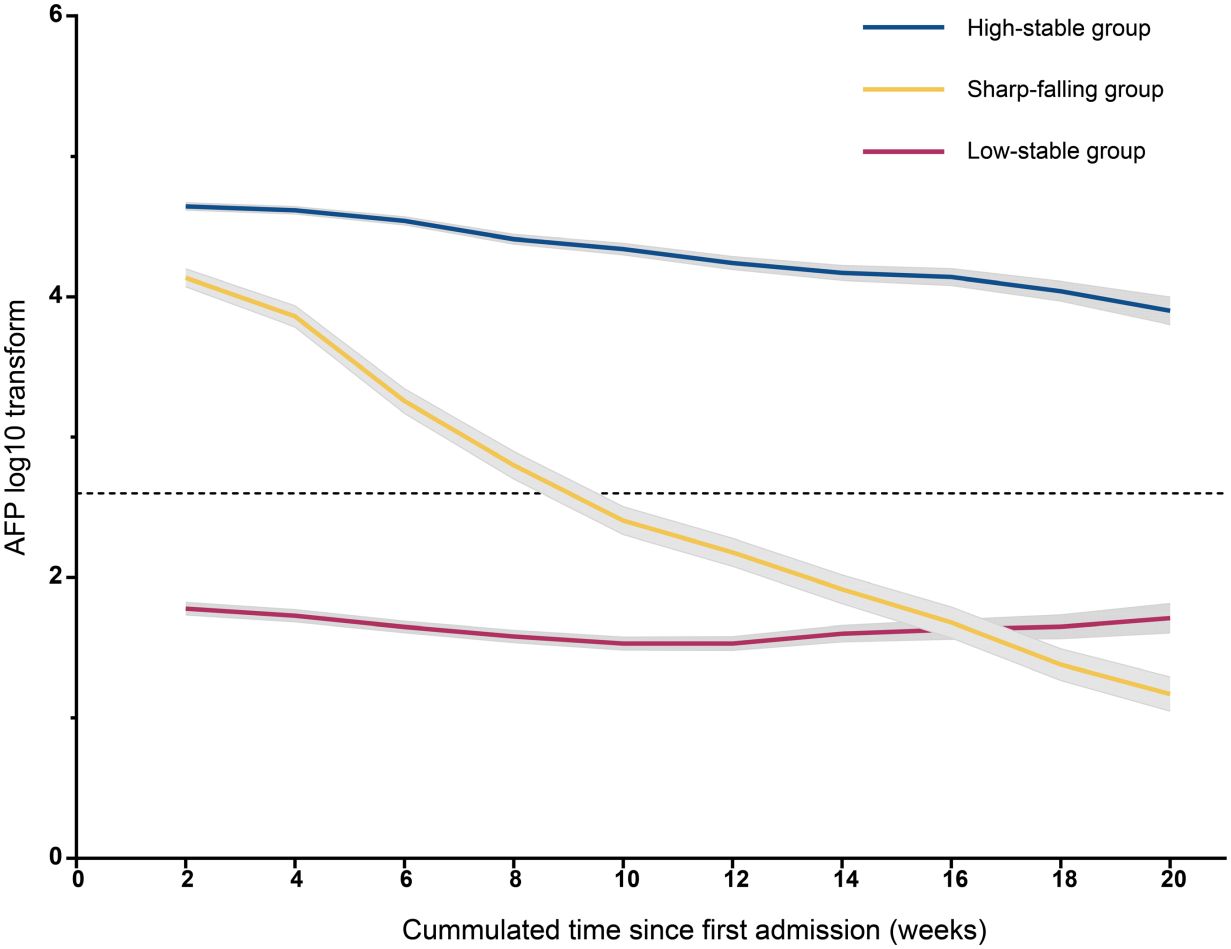
The trajectories of serum AFP in patients with large hepatocellular carcinoma after HAIC. Black dashed line: AFP value equaled to 400 ng/mL. Shadows: 95% confidence intervals. AFP, α‐fetoprotein; HAIC, hepatic arterial infusion chemotherapy; HCC, hepatocellular carcinoma.

The model fitting procedure for latent classes 1–5 of the linear, quadratic, and cubic curves using LCGMM is summarized in Table S2. According to the aforementioned criteria, a model of cubic parameters with three potential groups met the selection criteria best for log (AFP). Table S3 shows the results of the comprehensive parameter estimates using the best‐fitting three‐class quadratic curve. In addition, we evaluated the tangent slope of the AFP level at 1 week after HAIC and at approximately 1‐ to 4‐week intervals during the whole follow‐up period in the first 1 year (Table S4). Figure 2 shows the result of the class‐specific mean predicted serum AFP trajectory. The following three distinct class‐specific trajectories were estimated: high‐stability (37.0%; *n* = 373), sharp‐falling (15.7%; *n* = 159), and low‐stability trajectories (47.3%; *n* = 477). The AFP level remained within the range (400–1.21*10^5 ng/mL) with a slow downward tendency in the high‐stability group, with a significant difference between the first and last exam time points. In the sharp‐falling group, AFP fell sharply and quickly from a raised preoperative level (400–1.21 × 10^5^ ng/mL) toward the range of 0–400 ng/mL within 4 months of HAIC (*p* < 0.001), which could be defined as the AFP serological response curve. In the low‐stability group, the marker remained stable at low levels (AFP: 400 ng/mL) for 4 months after HAIC, with no significant increase or decrease.

### 3.3 Prognostic risk stratification of HCC patients with various trajectories

**FIGURE 3:**
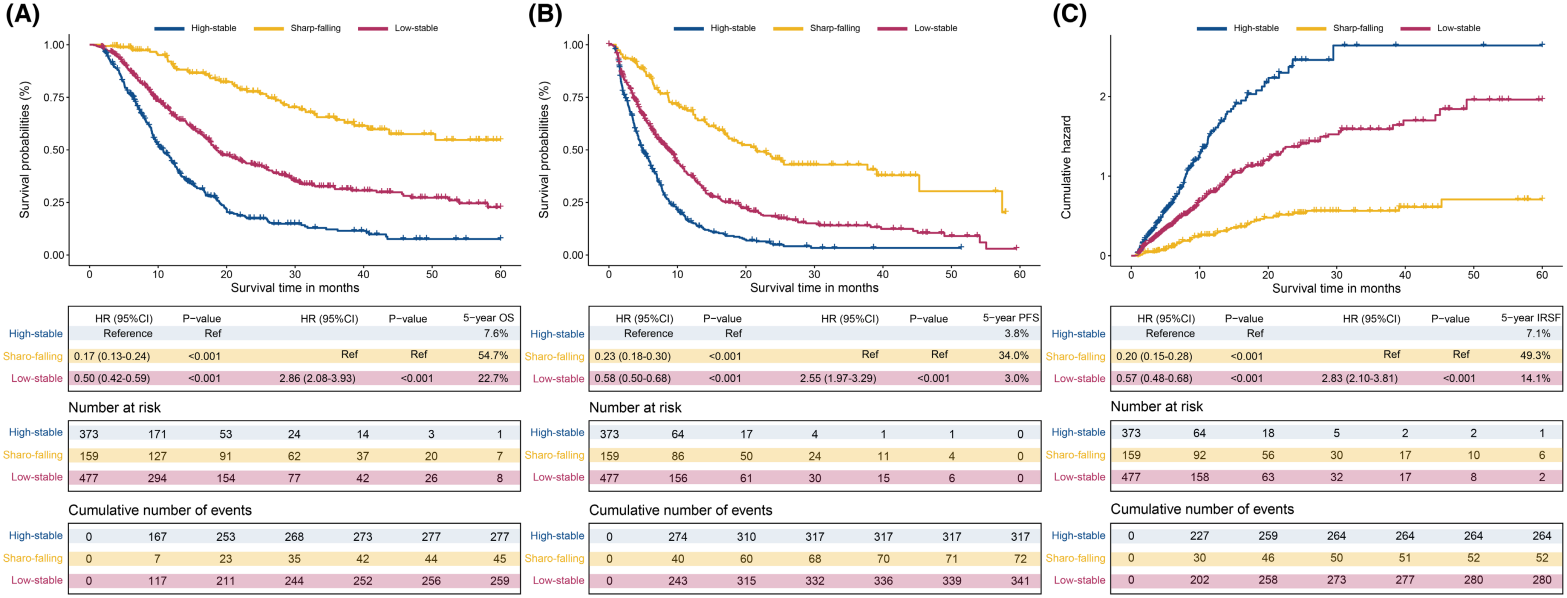
Comparing the survival among different AFP trajectory group. According to the LCGMM, HCC patients were divided into high‐stable, low‐stable, and sharp‐falling groups. Comparing the overall survival (OS) (A), progression‐free survival (PFS) (B) and intrahepatic recurrence‐free survival (IRFS) (C) among different AFP trajectories groups. *Note*: (A‐B) were developed with “pct” for survival probability in percentage; (C) was developed with “cumhaz” for the cumulative hazard function (f(y) = −log(y)). AFP, α‐fetoprotein; ALBI, Albumin‐Bilirubin; BCLC, Barcelona Clinic Liver Cancer; HAIC, hepatic arterial infusion chemotherapy; HCC, hepatocellular carcinoma; IRFS: Intrahepatic recurrence‐free survival; OS: Overall survival; PFS: Progression‐free survival.

Table 1 lists the baseline characteristics of the three trajectory groups after LCGMM analysis. The results suggested that subjects in the high‐stability group had higher ages, ALBI stage, more tumor number, ascites, vascular invasion, extrahepatic metastases and advanced BCLC stage than those in the low‐stability and sharp‐falling groups. Furthermore, subjects in the sharp‐falling group exhibited evidently higher values for age, ascites, ALBI stage, tumor number, vascular invasion, extrahepatic metastasis, and BCLC stage than those in the high‐stable and low‐stable groups, which indicated the relationship between these factors and the serum AFP trajectory. To further explore the role of these AFP trajectory groups on HCC patient prognosis, we evaluated the OS, PFS and IRFS for each trajectory group by survival and cumulative hazard curves (Figure 3, Figure S4). The results showed that HCC patients in the low‐stability group had higher OS rates than those in the high‐stability group (5‐year OS rate: 22.7% vs. 7.6%; HR, 0.17; 95% CI, 0.13–0.24; *p* < 0.001) but lower OS rates than those in the sharp‐falling group (5‐year OS rate: 54.7%) (Figure 3A). A similar difference was observed in predicting PFS; the 5‐year PFS rates for patients in the low‐stability group and high‐stability group (5‐year PFS rate: 3.0% vs. 3.8%; HR, 0.23; 95% CI, 0.18–0.30; *p* < 0.001; 5‐year IRFS rate: 7.1% vs. 14.1%; HR, 0.20; 95% CI, 0.15–0.28; *p* < 0.001) were lower than those in the low‐stability group (5‐year PFS rate: 34.0%, and 5‐year IRFS rate: 49.3%) (Figure 3B,C). Moreover, similar results were found with the Fine‐Gray competing risk regression analysis (Figure S4). The significant association between phenotype and clinical outcomes was verified by sensitivity analyses with the IPTW model, which helped to eliminate unmeasured confounding factors (Figure S5). The cumulative OS, PFS and IRFS were significantly different among the three groups (*p* < 0.001).

**TABLE 1:** Baseline characteristics of patients with large hepatocellular carcinoma after HAIC stratified by three types of AFP trajectory.

Variables	High‐stable *n* = 373	Sharp‐falling *n* = 159	Low‐stable *n* = 477	*p*‐Value
Age (years), *n* (%)				
≤65	316 (84.72)	105 (66.04)	337 (70.65)	<0.001
>60	57 (15.28)	54 (33.96)	140 (29.35)	
Gender, *n* (%)				
Female	48 (12.87)	19 (11.95)	43 (9.01)	0.181
Male	325 (87.13)	140 (88.05)	434 (90.99)	
ECOG score, *n* (%)				
0	353 (94.64)	149 (93.71)	455 (95.39)	0.691
1	20 (5.36)	10 (6.29)	22 (4.61)	
Comorbidity, *n* (%)				
Absence	342 (91.69)	137 (86.16)	399 (83.65)	0.002
Presence	31 (8.31)	22 (13.84)	78 (16.35)	
Etiology, *n* (%)				
Other	17 (4.56)	13 (8.18)	46 (9.64)	0.019
HBV	356 (95.44)	146 (91.82)	431 (90.36)	
Ascites, *n* (%)				
Absence	308 (82.57)	139 (87.42)	429 (89.94)	0.007
Presence	65 (17.43)	20 (12.58)	48 (10.06)	
ALBI stage, *n* (%)				
1	192 (51.47)	94 (59.12)	243 (50.94)	0.182
2 & 3	181 (48.53)	65 (40.88)	234 (49.06)	
Tumor size (cm), *n* (%)				
≤10	78 (20.91)	46 (28.93)	134 (28.09)	0.034
>10	295 (79.09)	113 (71.07)	343 (71.91)	
Tumor number, *n* (%)				
≤3	123 (32.98)	77 (48.43)	200 (41.93)	0.001
>3	250 (67.02)	82 (51.57)	277 (58.07)	
Vascular invasion, *n* (%)				
Absence	75 (20.11)	56 (35.22)	151 (31.66)	<0.001
Presence	298 (79.89)	103 (64.78)	326 (68.34)	
Metastasis, *n* (%)				
Absence	220 (58.98)	109 (68.55)	300 (62.89)	0.107
Presence	153 (41.02)	50 (31.45)	177 (37.11)	
BCLC stage, *n* (%)				
A	20 (5.36)	26 (16.35)	42 (8.81)	<0.001
B	41 (10.99)	22 (13.84)	77 (16.14)	
C	312 (83.65)	111 (69.81)	358 (75.05)	

*Note*: Differences are compared using the chi‐square test (or Fisher's exact test) for categorical measures.

Abbreviations: AFP, α‐fetoprotein; ALBI, Albumin‐Bilirubin; BCLC, Barcelona Clinic Liver Cancer; HAIC, hepatic arterial infusion chemotherapy; HCC, hepatocellular carcinoma.

### 3.4 The prognostic impact of classification pattern on HCC patients

**FIGURE 4:**
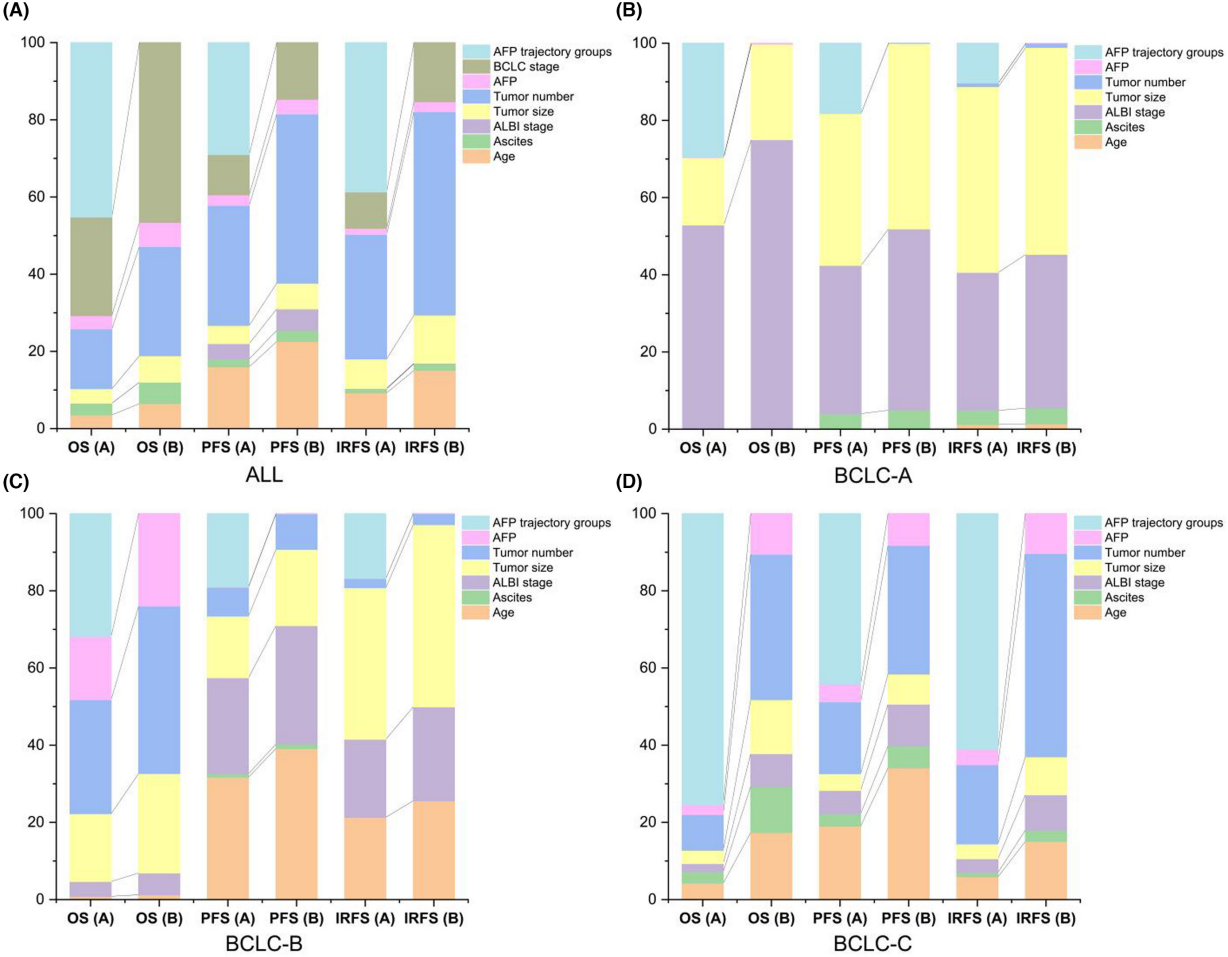
Relative importance of each risk factor for predicting the clinical outcomes of HCC patients. OS (A), PFS (A) and IRFS (A) consists of the relative importance of risk factors plus AFP trajectory groups; OS (B), PFS (B) and IRFS (B) only consists of the relative importance of risk factors. (A) the top three risk factor for all patients with large hepatocellular carcinoma after HAIC: OS (A) AFP trajectory groups, 45.2%; BCLC stage, 25.5%; tumor number, 15.5%; OS (B) BCLC stage, 46.6%; tumor number, 28.3%; tumor size, 6.8%; PFS (A) tumor number, 31.1%; AFP trajectory groups, 29.0%; age, 16.0%; PFS (B) tumor number, 43.8%; age, 22.5%; BCLC stage, 14.7%; IRFS (A) AFP trajectory groups, 38.7%; tumor number, 32.3%; BCLC stage, 9.4%; IRFS (B) tumor number, 52.7%; BCLC stage, 15.3%; age, 15.0%. (B) the top three risk factor for patients in BCLC‐A stage were ALBI stage, AFP trajectory groups and tumor size; (C) the top three risk factor for patients in BCLC‐B stage were AFP trajectory groups, tumor number and AFP level; (D) the top three risk factor for patients in BCLC‐C stage were AFP trajectory groups, tumor number and AFP level for OS. AFP, α‐fetoprotein; ALBI, Albumin‐Bilirubin; BCLC, Barcelona Clinic Liver Cancer; HAIC, hepatic arterial infusion chemotherapy; HCC, hepatocellular carcinoma; IRFS: Intrahepatic recurrence‐free survival; OS: Overall survival; PFS: Progression‐free survival.

Thereafter, we used univariable and multivariable Cox regression analyses to analyze the HRs and significant differences in clinical outcomes in each latent trajectory group. As shown in Table 2, the ALBI stage (HR: 1.19, 95% CI: 1.04–1.37; *p*‐value: 0.010), BCLC stage (HR: 2.02, 95% CI: 1.22–3.33; *p*‐value: 0.006), and APF trajectory (HR: 0.50, 95% CI: 0.38–0.66; *p*‐value: <0.001) were significant risk factors for predicting OS in multivariable Cox regression analysis. In addition, the APF trajectory groups (HR:0.61, 95% CI:0.52–0.72; *p*‐value: <0.001) were adjusted for age (HR: 0.82, 95% CI: 0.68–0.98; *p*‐value: 0.033), ALBI stage (HR:1.22, 95% CI: 1.08–1.37; *p*‐value: 0.001), BCLC stage (HR: 1.72, 95% CI: 1.19–2.47; *p*‐value: 0.004), tumor number (HR: 1.22, 95% CI: 1.03–1.44; *p*‐value:<0.001), and metastasis (HR: 1.30, 95% CI: 1.09–1.54; *p*‐value: 0.003) to predict PFS. Furthermore, we evaluated the relative contribution of each parameter to predict the clinical outcome, including age, ALBI stage, BCLC stage, tumor number, metastasis, and baseline AFP (Figure 4A). After adding the APF trajectory class to the abovementioned factors, we found that it was stronger than all these clinical parameters for OS, PFS, and IRFS (Figure 4A). In addition, we evaluated the relative contribution of these variables in the three BCLC stages. The APF trajectory class remained at its top location in all variables for the prediction of OS, PFS, and IRFS (Figure 4B–D).

**TABLE 2:** Risk Factors Associated with OS, PFS and IRFS of patients with HCC after HAIC according to Univariate and Multivariate Analysis.

Factors	Overall survival		Progression‐free survival		Intrahepatic recurrence‐free survival	
	Univariate Analysis	Multivariate Analysis	Univariate Analysis	Multivariate Analysis	Univariate Analysis	Multivariate Analysis
	HR (95 CI) & *p*‐Value	HR (95 CI) & *p*‐Value	HR (95 CI) & *p*‐Value	HR (95 CI) & *p*‐Value	HR (95 CI) & *p*‐Value	HR (95 CI) & *p*‐Value
Age (years)						
≤65	Ref	Ref	Ref	Ref	Ref	Ref
>60	**0.82 (0.67–0.99, 0.041)**	1.08 (0.88–1.32, 0.471)	**0.68 (0.57–0.82, <0.001)**	**0.82 (0.68–0.98, 0.033)**	**0.77 (0.64–0.93, 0.006)**	0.93 (0.76–1.13, 0.471)
Gender						
Female	Ref		Ref		Ref	
Male	1.03 (0.81–1.32, 0.810)		1.18 (0.93–1.49, 0.178)		1.24 (0.95–1.62, 0.120)	
ECOG score						
0	Ref		Ref		Ref	
1	0.94 (0.65–1.38, 0.763)		0.79 (0.55–1.12, 0.187)		0.82 (0.55–1.20, 0.302)	
Comorbidity						
Absence	Ref		Ref		Ref	
Presence	0.81 (0.63–1.05, 0.116)		0.86 (0.69–1.07, 0.184)		0.85 (0.67–1.08, 0.188)	
Etiology						
Other	Ref		Ref		Ref	
HBV	1.05 (0.76–1.45, 0.748)		1.01 (0.77–1.33, 0.938)		1.02 (0.75–1.38, 0.918)	
Ascites						
Absence	Ref	Ref	Ref	Ref	Ref	
Presence	**1.30 (1.02–1.65, 0.031)**	1.04 (0.81–1.32, 0.767)	**1.26 (1.02–1.57, 0.033)**	1.06 (0.85–1.32, 0.590)	1.16 (0.91–1.48, 0.234)	
ALBI stage						
1	Ref		Ref	Ref	Ref	Ref
2 & 3	**1.17 (1.04–1.33, 0.011)**	**1.19 (1.04–1.37, 0.010)**	**1.19 (1.07–1.32, 0.001)**	**1.22 (1.08–1.37, 0.001)**	**1.16 (1.03–1.30, 0.016)**	**1.18 (1.04–1.35, 0.012)**
Tumor size (cm)						
≤10	Ref		Ref	Ref	Ref	Ref
>10	**1.23 (1.02–1.49, 0.035)**	1.21 (0.99–1.47, 0.062)	**1.24 (1.05–1.48, 0.012)**	1.15 (0.97–1.37, 0.111)	**1.29 (1.07–1.56, 0.009)**	1.20 (0.99–1.46, 0.067)
Tumor number						
≤3	Ref		Ref	Ref	Ref	Ref
>3	**1.47 (1.24–1.74, <0.001)**	1.19 (0.99–1.43, 0.061)	**1.56 (1.34–1.81, <0.001)**	**1.22 (1.03–1.44, 0.019)**	**1.56 (1.32–1.85, <0.001)**	**1.22 (1.02–1.47, 0.032)**
Vascular invasion						
Absence	Ref		Ref	Ref	Ref	Ref
Presence	**1.63 (1.35–1.96, <0.001)**	0.89 (0.63–1.27, 0.526)	1.36 (1.15–1.60, <0.001)	1.02 (0.72–1.44, 0.927)	1.33 (1.11–1.59, 0.002)	1.09 (0.73–1.63, 0.659)
Metastasis						
Absence	Ref		Ref	Ref	Ref	Ref
Presence	**1.55 (1.31–1.83, <0.001)**	1.19 (0.99–1.44, 0.069)	**1.50 (1.29–1.74, <0.001)**	**1.30 (1.09–1.54, 0.003)**	**1.41 (1.19–1.66, <0.001)**	**1.25 (1.03–1.51, 0.021)**
BCLC stage						
A	Ref		Ref	Ref	Ref	Ref
B	**1.67 (1.11–2.50, 0.014)**	**1.31 (0.85–2.02, 0.226)**	**2.04 (1.45–2.86, <0.001)**	**1.72 (1.19–2.47, 0.004)**	**2.10 (1.45–3.04, <0.001)**	**1.75 (1.17–2.61, 0.006)**
C	**2.60 (1.83–3.68, <0.001)**	**2.02 (1.22–3.33, 0.006)**	**2.20 (1.64–2.95, <0.001)**	**1.47 (0.93–2.34, 0.101)**	**2.12 (1.54–2.93, <0.001)**	**1.36 (0.81–2.30, 0.245)**
AFP trajectory						
High‐stable	Ref		Ref	Ref	Ref	Ref
Sharp‐falling	**0.18 (0.13–0.24, <0.001)**	**0.19 (0.13–0.26, <0.001)**	**0.23 (0.18–0.30, <0.001)**	**0.25 (0.19–0.33, <0.001)**	**0.20 (0.15–0.27, <0.001)**	**0.22 (0.16–0.30, <0.001)**
Low‐stable	**0.49 (0.42–0.59, <0.001)**	**0.50 (0.38–0.66, <0.001)**	**0.58 (0.49–0.67, <0.001)**	**0.61 (0.52–0.72, <0.001)**	**0.57 (0.48–0.67, <0.001)**	**0.59 (0.50–0.71, <0.001)**

*Note*: Cox regression analyses are used to calculate the hazard ratios (HRs) and 95% confidence intervals (CIs) based on overall survival (OS), progression‐free survival (PFS) and intrahepatic recurrence‐free survival (IRFS). Covariables that are significant in univariable Cox regression analysis (*p* < 0.05) are included in the multivariable analysis. The bold values represent this variable was a risk factor for survival outcomes.

Abbreviations: ALBI, Albumin‐Bilirubin; CI, confidence interval; HAIC, hepatic arterial infusion chemotherapy; HCC, hepatocellular carcinoma; HRs, hazard ratios; IRFS, Intrahepatic recurrence‐free survival; OS, Overall survival; PFS, Progression‐free survival.

### 3.5 Sensitivity analyses

**FIGURE 5:**
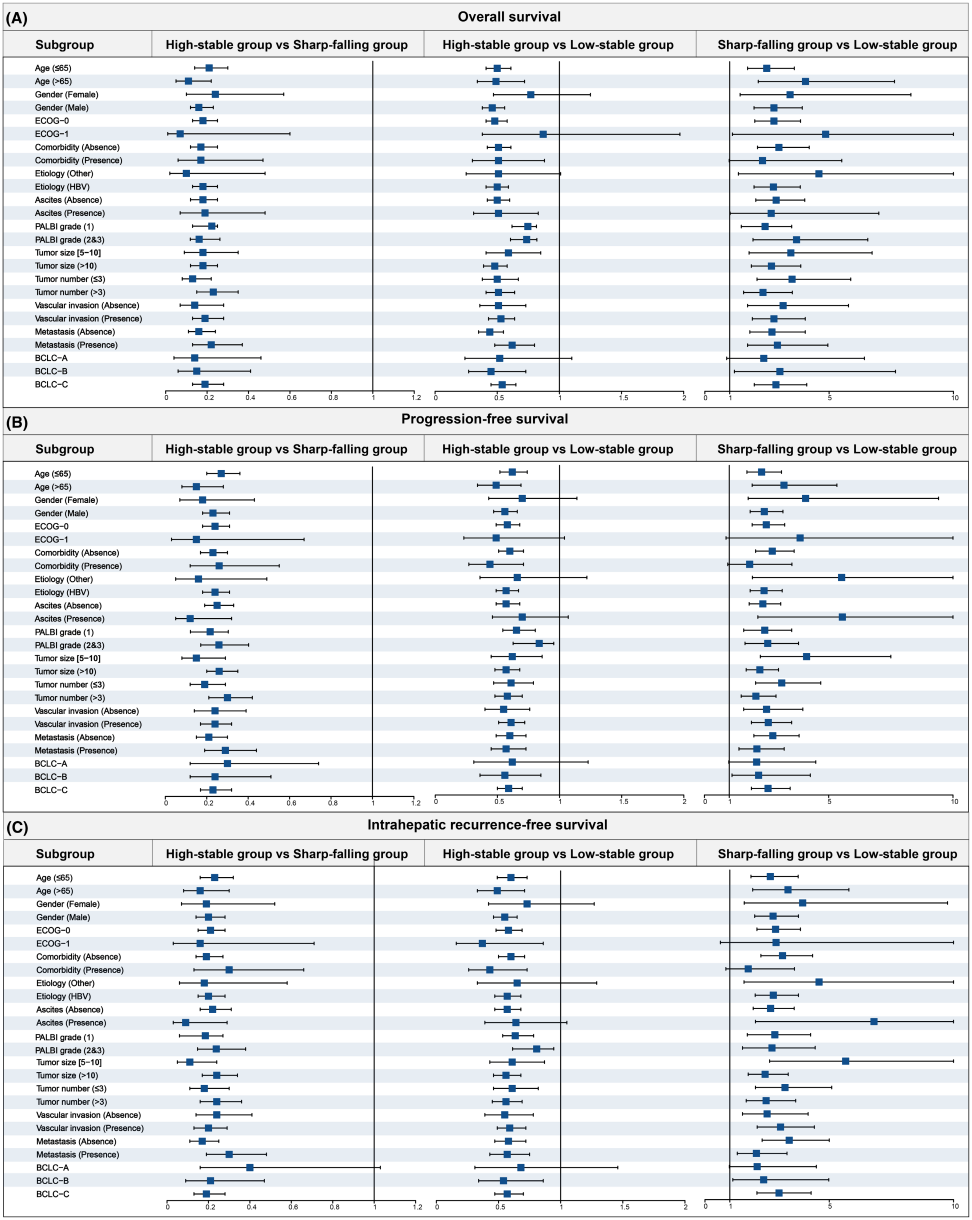
Subgroup analyses of prognosis for serum AFP trajectories stratified by clinical factors. (A) for OS, (B) for PFS, and (C) for IRFS. AFP, α‐fetoprotein; ALBI, Albumin‐Bilirubin; BCLC, Barcelona Clinic Liver Cancer; HAIC, hepatic arterial infusion chemotherapy; HCC, hepatocellular carcinoma; IRFS: Intrahepatic recurrence‐free survival; OS: Overall survival; PFS: Progression‐free survival.

The significant association between phenotype and clinical outcomes was verified by sensitivity analyses with the IPTW model, which helped to eliminate unmeasured confounding factors (Table S5, Figure S5). The cumulative OS was significantly different among the three groups (*p* < 0.001). According to HCC patient characteristics, exploratory subgroup analyses are shown in Table 3 and Table S6. Three forest plots were used to outline all subgroup risk factors for OS, PFS and IRFS (Figure 5A–C **)**. These subgroup analyses of OS, PFS and IRFS showed similar results to those for the overall population. Compared with the low‐stability group, the high‐stability group had a significantly higher HR in the subgroup, and the HCC patients in the sharp‐falling group were found to have a HAIC response with the best prognosis. Similar results were also found in different BCLC stages (Figure S6).

**TABLE 3:** Subgroup analysis of overall survival, progression‐free survival and intrahepatic recurrence‐free survival for serum AFP trajectories stratified by clinical features.

Subgroups	High‐stable vs. Low‐stable group			Sharp‐falling vs. Low‐stable group		
	OS	PFS	IRFS	OS	PFS	IRFS
	HR (95 CI) & *p*	HR (95 CI) & *p*	HR (95 CI) & *p*	HR (95 CI) & *p*	HR (95 CI) & *p*	HR (95 CI) & *p*
Age ≤ 65 years	0.50 (0.41–0.61, <0.001)	0.62 (0.52–0.74, <0.001)	0.60 (0.49–0.73, <0.001)	2.49 (1.72–3.60, <0.001)	2.30 (1.71–3.10, <0.001)	2.63 (1.85–3.75, <0.001)
Age > 65 years	0.49 (0.34–0.72, <0.001)	0.49 (0.34–0.69, <0.001)	0.49 (0.33–0.71, <0.001)	4.05 (2.15–7.63, <0.001)	3.20 (1.92–5.33, <0.001)	3.34 (1.92–5.79, <0.001)
Gender (Female)	0.77 (0.47–1.25, 0.287)	0.70 (0.43–1.14, 0.151)	0.73 (0.42–1.27, 0.263)	3.43 (1.42–8.29, 0.006)	4.07 (1.76–9.42, 0.001)	3.93 (1.58–9.76, 0.003)
Gender (Male)	0.46 (0.38–0.56, <0.001)	0.56 (0.47–0.66, <0.001)	0.55 (0.46–0.65, <0.001)	2.78 (1.98–3.92, <0.001)	2.41 (1.84–3.16, <0.001)	2.74 (2.00–3.76, <0.001)
Etiology (Other)	0.51 (0.25–1.01, 0.054)	0.66 (0.36–1.22, 0.187)	0.65 (0.33–1.29, 0.222)	4.59 (1.35–15.62, 0.015)	5.52 (1.93–15.84, 0.001)	4.59 (1.57–13.39, 0.005)
Etiology (HBV)	0.50 (0.41–0.59, <0.001)	0.57 (0.49–0.67, <0.001)	0.57 (0.47–0.68, <0.001)	2.76 (1.98–3.84, <0.001)	2.40 (1.84–3.13, <0.001)	2.75 (2.02–3.76, <0.001)
ALBI stage 1 & 2	0.50 (0.42–0.59, <0.001)	0.56 (0.48–0.66, <0.001)	0.57 (0.48–0.68, <0.001)	2.82 (2.04–3.89, <0.001)	2.56 (1.96–3.34, <0.001)	2.81 (2.07–3.81, <0.001)
ALBI stage 3	0.21 (0.06–0.77, 0.019)	0.45 (0.14–1.41, 0.168)	0.23 (0.06–0.83, 0.024)	4.82 (0.60–38.53, 0.138)	2.87 (1.07–7.73, 0.037)	3.76 (0.82–17.31, 0.089)
Tumor size ≤10 cm	0.59 (0.41–0.85, 0.004)	0.62 (0.45–0.86, 0.004)	0.61 (0.43–0.87, 0.007)	3.46 (1.78–6.73, <0.001)	4.11 (2.25–7.50, <0.001)	5.66 (2.60–12.29, <0.001)
Tumor size >10 cm	0.48 (0.39–0.58, <0.001)	0.57 (0.48–0.68, <0.001)	0.56 (0.46–0.68, <0.001)	2.68 (1.87–3.86, <0.001)	2.23 (1.68–2.98, <0.001)	2.42 (1.74–3.35, <0.001)
Tumor number ≤3	0.50 (0.38–0.67, <0.001)	0.61 (0.47–0.79, <0.001)	0.61 (0.46–0.82, 0.001)	3.51 (2.10–5.87, <0.001)	3.11 (2.06–4.68, <0.001)	3.22 (2.03–5.10, <0.001)
Tumor number >3	0.51 (0.41–0.64, <0.001)	0.58 (0.48–0.70, <0.001)	0.56 (0.45–0.69, <0.001)	2.34 (1.56–3.52, <0.001)	2.07 (1.48–2.88, <0.001)	2.46 (1.66–3.65, <0.001)
Vascular invasion (Absence)	0.51 (0.36–0.73, <0.001)	0.55 (0.40–0.76, <0.001)	0.55 (0.39–0.78, 0.001)	3.15 (1.72–5.78, <0.001)	2.50 (1.58–3.96, <0.001)	2.50 (1.51–4.15, <0.001)
Vascular invasion (Presence)	0.53 (0.43–0.64, <0.001)	0.61 (0.51–0.72, <0.001)	0.59 (0.49–0.72, <0.001)	2.78 (1.91–4.04, <0.001)	2.57 (1.89–3.51, <0.001)	3.04 (2.10–4.40, <0.001)
Metastasis (Absence)	0.44 (0.35–0.55, <0.001)	0.60 (0.49–0.73, <0.001)	0.58 (0.47–0.72, <0.001)	2.70 (1.81–4.04, <0.001)	2.75 (1.99–3.81, <0.001)	3.39 (2.30–5.00, <0.001)
Metastasis (Presence)	0.62 (0.48–0.80, <0.001)	0.57 (0.45–0.73, <0.001)	0.57 (0.43–0.75, <0.001)	2.92 (1.72–4.95, <0.001)	2.11 (1.39–3.21, <0.001)	2.07 (1.30–3.30, 0.002)
BCLC‐A stage	0.52 (0.24–1.10, 0.088)	0.62 (0.31–1.23, 0.171)	0.68 (0.31–1.46, 0.319)	2.37 (0.88–6.42, 0.088)	2.10 (0.98–4.48, 0.055)	2.10 (0.98–4.48, 0.055)
BCLC‐B stage	0.45 (0.27–0.73, 0.001)	0.56 (0.36–0.85, 0.007)	0.54 (0.34–0.86, 0.009)	3.02 (1.19–7.67, 0.020)	2.18 (1.11–4.26, 0.023)	2.36 (1.12–4.98, 0.024)
BCLC‐C stage	0.54 (0.45–0.65, <0.001)	0.59 (0.50–0.70, <0.001)	0.57 (0.47–0.70, <0.001)	2.86 (1.99–4.10, <0.001)	2.56 (1.89–3.45, <0.001)	2.98 (2.09–4.27, <0.001)

*Note*: The subgroup analysis was developed with Cox regression analyses based on OS, PFS and IRFS, which were noted in Stable 6–8.

Abbreviations: ALBI, Albumin‐Bilirubin; CI, confidence interval; HAIC, hepatic arterial infusion chemotherapy; HCC, hepatocellular carcinoma; HRs, hazard ratios; IRFS: Intrahepatic recurrence‐free survival; OS: Overall survival; PFS: Progression‐free survival.

## 4 DISCUSSION

α‐fetoprotein plays an important role in detecting and monitoring HCC. There is an increasing number of serum tumor marker‐based prediction models derived from AFP, which provide meaningful help and guidance for clinicians in diagnosis, decision‐making, and disease management. Among them, the GALAD model,^27^ including gender, age, AFP, and des‐gamma‐carboxy prothrombin (DCP), has a confirmed high sensitivity and screening‐level specificity for outcome prediction in most countries. In various interventional modalities of HCC, these tumor marker‐based models have also been applied for the prediction of postoperative recurrence or death. For example, Jeongin Yoo et al. established a serum tumor marker‐based MoRAL score for predicting recurrence after RFA of very‐early/early‐stage HCC.^28^ Linbin Lu et al. divided the AFP trajectories into three groups, and the trajectories of serum AFP levels based on an LCGMM were used to examine their impact on the survival outcomes of patients with HCC who underwent TACE.^21^

HAIC has always been an effective and safe interventional treatment for large HCC.^29^ To our knowledge, a noninvasive method for predicting and monitoring recurrence and death after HAIC treatment has not been established. In this study, we proposed a novel method to predict the outcome after HAIC treatment by using dynamic AFP trajectories and summarized and classified different AFP trajectories, which can provide more guidance to help interventional radiologists during HAIC treatment and provide screening paths for the design of clinical trials. In this study, the median number of HAIC sessions was four. Due to the formulation and implementation of the 21‐day repeat chemotherapy infusion regimen, the sessions of AFP detection increased, and the interval time decreased. As a result, more intensive AFP trajectories were created, which were divided into three types, including the high‐stability, low‐stability, and sharp‐falling types, based on an LCGMM. In the three types of AFP trajectories, the ORR in the sharp‐falling group was significantly higher than that in the other groups, especially the high‐stability group. Among them, the sample size of the low‐stability AFP trajectory was the smallest, which also suggests that AFP levels vary greatly during HAIC treatment for large HCC. This was different from the sample size of the low‐stability AFP trajectory in a previous report of TACE treatment in HCC.^21^ This also proved the higher ORR of patients who received HAIC than TACE.

Previously, numerous studies reported that the change in AFP levels before and after targeted chemotherapy plays a crucial role in the therapeutic response.^30^, ^31^ Therefore, AFP decline was the robust subclass across all stages of HCC, which supported the finding of AFP serological response and implied a specified pathophysiological process. Linbin Lu et al. proposed the application of AFP trajectories for the prediction of outcomes of patients with intermediate‐stage HCC who underwent TACE,^21^ which has been confirmed by the above hypothesis. Compared with TACE, HAIC is easy to perform and has better repeatability due to fewer complications related to HAIC operation and AEs than TACE. Accordingly, this cohort of patients treated with HAIC had a better ORR than those treated with TACE. Interestingly, we also found similar survival outcomes among the three different AFP trajectory groups in this study. After IPTW, these outcomes remained unchanged. Different from the former study, the cutoff value of AFP proposed by us was 400 ng/mL instead of 25 ng/mL, which may be due to the higher level of AFP caused by the stronger tumor activity of large HCC in advanced stages.

To explore the sensitivity of predicting oncological outcomes after HAIC treatment, we used different statistical methods to prove the robustness of the AFP trajectory classification. In this study, different statistical methods, including IPTW, subgroup and multivariable Cox regression analyses, all showed that the dynamic AFP trajectory is superior to preoperative AFP in indicating death and recurrence after HAIC treatment. Especially for predicting the death outcome, the AFP trajectory has the best predictive ability among many variables. However, for tumor progression after HAIC, the number of tumors is also a very important risk factor, and the discrimination is superior to the AFP trajectory and preoperative AFP level, which requires physicians to be alert before HAIC treatment. Furthermore, we also verified the effectiveness of AFP trajectories in BCLC A, B and C stages. The survival curve comparison between different groups in the three BCLC stages remained statistically significant. The predictive ability of the dynamic AFP trajectory yielded the optimal predictive performance among all variables in BCLC A, B, and C. These results indicated that the AFP trajectory presents high‐level sensitivity and discrimination of the survival outcome regardless of BCLC stage. In addition, we observed that the HRs for death and disease progression in the BCLC A group were lower than those in the BCLC B or C groups, which suggested an interaction between the dynamic AFP trajectory and the three BCLC stages.

AFP may accelerate the proliferation of HCC cells by interacting with the AFP receptor (AFPR), leading to the activation of the phosphatidylinositol 3‐kinase (PI3K), protein kinase B in the serine/threonine protein kinases (AKT) and mammalian target of rapamycin (mTOR) pathways.^32^, ^33^ This interaction results in the high expression of vascular endothelial growth factor (VEGF), an important mediator in hepatocarcinogenesis, by stimulating new blood vessel formation, which leads to HCC invasion and metastasis.^34^ The dynamic AFP trajectory represents the expression of HCC biological behavior and aggressiveness, and the change in AFP levels at different time points in the course of HAIC treatment can better reflect the prediction of such oncological behavior and outcome. However, the mechanisms of these interactions found in the sensitivity analysis remain unclear and need to be explored in further research. In practice, the dynamic AFP trajectory may be utilized to identify high‐risk patients who cannot be recognized only by clinicopathological features and to provide them with targeted care and prompt treatment.

This study also had several limitations. First, although three types of AFP trajectories covered most of the enrolled patients in this study, there were still a small number of HCC patients who were not suitable for the above three types, which may affect the final survival outcome to a certain extent. Second, previous studies have shown that the degree of pathological differentiation determines the effect of chemotherapy response, but this study did not collect pathological differentiation as a clinical variable. Therefore, this factor may also bring some interaction and confusion to the treatment effect and survival outcome of the AFP trajectory groups. Third, our three proposed AFP trajectories were based on the Chinese population with prevalent hepatitis B infection and cirrhosis. The nonproliferation class of HCC, more commonly with HCV infection and alcohol abuse in foreign populations, may have different AFP trajectories. Finally, the number of patients in BCLC stage A remains relatively low; thus, our conclusions might not be suitable for early‐stage HCC with a high tumor burden. Moreover, bias could be caused by residual and unmeasured confounders. Our findings must also be verified by a prospective randomized controlled trial and a larger‐scale population.

In summary, three distinct AFP trajectories were identified for patients with large HCC who received HAIC treatment. Accordingly, different survival outcomes were presented in the three AFP trajectory groups and three subgroups of BCLC stages. These study results imply that HCC patients with sharp‐falling AFP trajectories have optimal survival outcomes after multiple‐cycle HAIC, which provides a potential monitoring tool for improving clinical decision‐making.

## AUTHOR CONTRIBUTIONS

**Chao An:** Writing – original draft (equal). **Ran Wei:** Methodology (lead). **Wang Yao:** Resources (equal). **Wenwen Han:** Formal analysis (equal). **Wang Li:** Software (equal). **Ge Shi:** Writing – review and editing (equal). **Peihong Wu:** Writing – review and editing (equal).

## FUNDING INFORMATION

His work was supported by the National Natural Science Fund of China (Grant No. 8187146, 82202282). The funders had no role in study design, data collection and analysis, decision to publish, or preparation of the manuscript.

## CONFLICT OF INTEREST STATEMENT

No potential conflicts of interest were disclosed.

## ETHICS STATEMENT

This retrospective study was approved by the Institutional Review Board of Sun Yat‐sen University Cancer Center (B2022‐11‐01) and was conducted following the principles of the Declaration of Helsinki. The requirement for written informed consent was waived because of the retrospective nature of the study. The key raw data of this study were uploaded to the Research Data Deposit database (www.researchdata.org.cn. RDD2021002019).

## CONSENT TO PARTICIPATE

Yes.

## Supporting information


Appendix S1.

## Data Availability

The in‐house developed medical database of this study is publicly accessible at: http://www.yunedc.cn/#/login.
