# Supplementary material for: Association of serum AFP trajectories and hepatocellular carcinoma outcomes after hepatic arterial infusion chemotherapy: A longitudinal, multicenter study
Source: Cancer Med. 2024 May 31;13(11):e7319. doi: 10.1002/cam4.7319 (PMC11141330; doi:10.1002/cam4.7319)
Supplement: Supplementary file 1 — Appendix S1. [file CAM4-13-e7319-s001.doc]

**Association of Serum AFP Trajectories and Hepatocellular Carcinoma Outcomes after Hepatic Arterial Infusion Chemotherapy: A Longitudinal, Multicenter Study**

**This supplementary material includes:**

1. **Supplementary Methods**

**E1.1** HAIC procedure

**E1.2** HAIC using FOLFOX regimen

**E1.3** Criteria for protocol treatment discontinuation

**E1.4** Clinical data selection

1. **Supplementary Tables**

**Table S1.** Baseline characteristics of patients with large hepatocellular carcinoma after HAIC in these cohort.

**Table S2.** Latent Class Growth Mixture models (LCGMM) results of the model fitting process.

**Table S3.** Parameter estimates for the best fitting 3-class quadratic latent class growth mixture model fitted to the α-fetoprotein (AFP) data.

**Table S4.** Estimated slope and value of AFP in different points by 3-class quadratic LCGMM model.

**Table S5.** The coefficients of HCC patients among different groups after inverse-probability-of-treatment weighted analysis (IPTW)

**Table S6.** Subgroup analysis of overall survival, progression-free survival and intrahepatic recurrence-free survival between High-stable and Sharp-falling groups by clinical features

1. **Supplementary Figures**

**Figure. S1** An example of patients with large HCC receiving HAIC plus lenvatinib. After four cycles of HAIC, the large HCC shrank significantly (the tumor diameter from 12.8 cm to 7.5 cm), and then one month after HAIC treatment, the tumor thrombus disappeared with oral lenvatinib.

**Figure. S2** An example of patients with large HCC receiving HAIC plus PD-1. After six cycles of HAIC, the large HCC shrank significantly (the tumor diameter from 11.4 cm to 4.8 cm), and then one day after HAIC treatment, the tumor thrombus disappeared when PD-1 was infused intravenously

**Figure. S3** An example of patients with large HCC receiving HAIC combined with sequential RFA. After four cycles of HAIC, the large HCC shrank significantly (the tumor diameter from 5.2 cm to 2.3cm, nodule with yellow arrow), and then 42 days after HAIC treatment RFA was used, eradicating the large HCC with complete ablation.

**Figure. S4** Comparisons of cumulative incidence of intrahepatic recurrence-free survival (IRFS) among patients in different AFP trajectories groups. (A) for IRFS; (B) for a non- IRFS or all-cause death were considered competing events. P values were calculated with the Gray test

**Abbreviations:** AFP, a-fetoprotein; BCLC, Barcelona Clinic Liver Cancer; ALBI, Albumin Bilirubin; HCC, hepatocellular carcinoma; HAIC, hepatic arterial infusion chemotherapy; IRFS: Intrahepatic recurrence-free survival; PFS: Progression-free survival; OS: Overall survival.

**Figure. S5** The inverse-probability-of-treatment weighted analysis (IPTW) for HCC patients after HAIC in different BCLC stages. Comparing the overall survival (OS)(A), progression-free survival (PFS) (B) and intrahepatic recurrence-free survival (IRFS) (C) among different AFP Trajectories groups of BCLC A stage. The patients in different groups were adjusted with age, etiology, comorbidity, ALBI stage, tumor size, Ascites, tumor number, vascular invasion and BCLC stage.

**Abbreviations:** AFP, a-fetoprotein; BCLC, Barcelona Clinic Liver Cancer; ALBI, Albumin Bilirubin; HCC, hepatocellular carcinoma; HAIC, hepatic arterial infusion chemotherapy; IRFS: Intrahepatic recurrence-free survival; PFS: Progression-free survival; OS: Overall survival.

**Figure. S6** Subgroup analysis of HCC patients after HAIC in different BCLC stages. Comparing the overall survival (OS) (A), progression-free survival (PFS) (B) and intrahepatic recurrence-free survival (IRFS) (C) among different AFP trajectories groups of BCLC A stage. Comparing the OS (D), PFS (E) and IRFS (F) among different AFP Trajectories groups of BCLC B stage. Comparing the OS (G), PFS (H) and IRFS (I) among different AFP Trajectories groups of BCLC C stage.

**Abbreviations:** AFP, a-fetoprotein; BCLC, Barcelona Clinic Liver Cancer; ALBI, Albumin Bilirubin; HCC, hepatocellular carcinoma; HAIC, hepatic arterial infusion chemotherapy; IRFS: Intrahepatic recurrence-free survival; PFS: Progression-free survival; OS: Overall survival.

1. **Supplementary Methods**

**E1.1** **HAIC procedure**

HAIC procedures have been described in a serial of previous studies ^[1-2]^. All procedures were performed using digital subtraction angiography (Philips, type FD 20 1250 mA, Amsterdam, Netherlands). The artery sheath catheter was inserted into the femoral artery using the modified Seldinger technique. A 5-Fr Yashiro catheter (Terumo, Tokyo, Japan) was advanced into the celiac trunk and superior mesenteric artery to assess the feeding hepatic artery. A 2.7-Fr micro-catheter (Terumo, Tokyo, Japan) was inserted in the feeding artery. If the tumors simultaneously accepted blood supply from the celiac trunk and superior mesenteric artery, the microcatheter would be placed into the largest tumor feeding arteries. The peripheral end of the micro-catheter would be locked with a heparin lock (10 ml, 10,000 units, 1: 1,000 dilution) to prevent clotting of the catheter. The peripheral part of the catheter exposed outside the body would be covered by medical sterile gauze and fastened on the skin of the thigh using medical rubberized fabric and a bandage.

**E1.2 HAIC using** **FOLFOX regimen**

All chemo-drugs were given by hepatic arterial infusion through the micro-catheter. A modified FOLFOX6 regimen ^[3-5]^, including oxaliplatin (130 mg/m^2^ infusion for 3 h on day 1), leucovorin (200 mg/m^2^ for 3–5 hours on day 1), and Fluorouracil (400 mg/m2 in bolus, and then 2,400 mg/m^2^ continuous infusion 46 h) was applied. This 3-week period constituted one cycle, and cycles would be repeated until discontinuation (intrahepatic lesions progressed or toxicity became unacceptable) of the protocol treatment.

**E1.3 Criteria for protocol treatment discontinuation**

1. Tumor progression

The progression disease (PD) was assessed by dynamic CT or MRI based on modified Response Evaluation Criteria in Solid Tumor (mRECIST).

1. Intolerable adverse event

i) Patient could not resume HAIC after 30 days of interruption due to an adverse event;

ii) An adverse event meeting the criteria for HAIC dose reduction occurred after the dose was already reduced to the lowest level;

iii) Life-threatening adverse event;

C)The need for another anticancer treatment due to downstaging at the physician’ s discretion;

D) HAIC became technically infeasible;

E) Patient requested discontinuation of the study;

F) Death.

**E1.4 Clinical data selection**

Clinical data including age (≤65 years，>65 years), gender (male, female), ECOG status (0,1), comorbidities, cirrhosis, ascites, cause of liver disease (HBV, hepatitis B virus; HCV, hepatitis C virus; alcoholism; others), the largest diameter of tumor, tumor number, macroscopic portal vein invasion and extrahepatic metastasis. Laboratory findings included AFP, serum albumin [ALB], total bilirubin [TB], platelet counts, prothrombin time (PT), international normalized ratio [INR], aspartate aminotransferase [AST] and alanine aminotransferase [ALT], C reactive protein [CRP], creatinine, neutrophils and lymphocytes. ALBI and PALBI grades were used to replace CTP grade for their objectiveness. ALBI score was calculated before treatment using the appropriate clinical parameters and ALBI grade was defined as follows: (log 10 bilirubin [BI] [μ mol /L] × 0.66) + (albumin [AL] [g/L] ×-0.085), (grade 1, 2, and 3 = ≤ -2.60, > -2.60 to -1.39, and > -1.39, respectively).

**Reference:**

1. Ueshima K, Komemushi A, Aramaki T, Iwamoto H, Obi S, Sato Y, Tanaka T, Matsueda K, Moriguchi M, Saito H, Sone M, Yamagami T, Inaba Y, Kudo M, Arai Y. Clinical Practice Guidelines for Hepatic Arterial Infusion Chemotherapy with a Port System Proposed by the Japanese Society of Interventional Radiology and Japanese Society of Implantable Port Assisted Treatment. Liver Cancer. 2022 May 5;11(5):407-425. doi: 10.1159/000524893.

2. Yamasaki T, Saeki I, Yamauchi Y, Matsumoto T, Suehiro Y, Kawaoka T, Uchikawa S, Hiramatsu A, Aikata H, Kobayashi K, Kondo T, Ogasawara S, Chiba T, Takami T, Chayama K, Kato N, Sakaida I. Management of Systemic Therapies and Hepatic Arterial Infusion Chemotherapy in Patients with Advanced Hepatocellular Carcinoma Based on Sarcopenia Assessment. Liver Cancer. 2022 Feb 22;11(4):329-340. doi: 10.1159/000522389.

3. Wang T, Dong J, Zhang Y, Ren Z, Liu Y, Yang X, Sun D, Wang Y. Efficacy and safety of hepatic artery infusion chemotherapy with mFOLFOX in primary liver cancer patients with hyperbilirubinemia and ineffective drainage: a retrospective cohort study. Ann Transl Med. 2022 Apr;10(7):411. doi: 10.21037/atm-22-978.

4. Lai Z, Huang Y, Wen D, Lin X, Kan A, Li Q, Wei W, Chen M, Xu L, He M, Shi M. One day versus two days of hepatic arterial infusion with oxaliplatin and fluorouracil for patients with unresectable hepatocellular carcinoma. BMC Med. 2022 Oct 31;20(1):415. doi: 10.1186/s12916-022-02608-6.

5. Si T, Huang Z, Khorsandi SE, Ma Y, Heaton N. Hepatic arterial infusion chemotherapy versus transarterial chemoembolization for unresectable hepatocellular carcinoma: A systematic review with meta-analysis. Front Bioeng Biotechnol. 2022 Sep 27;10:1010824. doi: 10.3389/fbioe.2022.1010824.

1. **Supplementary Tables**

**Table S1.** Baseline characteristics of patients with large hepatocellular carcinoma after HAIC in these cohort.

| **Variables** | **Patients who received HAIC**  **n=1009** |
| --- | --- |
| **Age (years), n (%)** |  |
| ≤65 | 758 (75.12) |
| > 60 | 251 (24.88) |
| **Gender, n (%)** |  |
| Female | 110 (10.90) |
| Male | 899 (89.10) |
| **ECOG score, n (%)** |  |
| 0 | 957 (94.85) |
| 1 | 52 (5.15) |
| **Comorbidity, n (%)** |  |
| Absence | 878 (87.02) |
| Presence | 131 (12.98) |
| **Etiology, n (%)** |  |
| Other | 76 (7.53) |
| HBV | 933 (92.47) |
| **Ascites** |  |
| Absence | 876 (86.82) |
| Presence | 133 (13.18) |
| **ALBI stage, n (%)** |  |
| 1 | 529 (52.5) |
| 2&3 | 480 (47.5) |
| **Tumor size (cm), n (%)** |  |
| ≤10 | 258 (25.57) |
| >10 | 751 (74.43) |
| **Tumor number, n (%)** |  |
| ≤3 | 400 (39.64) |
| >3 | 609 (60.36) |
| **Vascular invasion, n (%)** |  |
| Absence | 282 (27.95) |
| Presence | 727 (72.05) |
| **Metastasis, n (%)** |  |
| Absence | 629 (62.34) |
| Presence | 380 (37.66) |
| **BCLC stage, n (%)** |  |
| A | 88 (8.72) |
| B | 140 (13.88) |
| C | 781 (77.40) |
| **Follow-up time (mean-SD)** | 17.02 (14.07) |

**Abbreviations:** AFP, a-fetoprotein; BCLC, Barcelona Clinic Liver Cancer; ALBI, Albumin Bilirubin; HCC, hepatocellular carcinoma; HAIC, hepatic arterial infusion chemotherapy.

**Table S2. Latent Class Growth Mixture models (LCGMM) results of the model fitting process.**

| **No. of latent classes** | **Polynomial degree** | **AIC** | **BIC** | **Patients Per class (%)** | **Mean posterior probabilities** |
| --- | --- | --- | --- | --- | --- |
| 1 | Linear | 14897.77 | 14917.43 | NA | NA |
|  | Quadratic | 9326.85 | 9366.19 | NA | NA |
|  | Cubic | 9327.54 | 9371.79 | NA | NA |
| 2 | Linear | 11032.04 | 11071.37 | 9.9/90.1 | 1.00/0.98 |
|  | Quadratic | 10803.02 | 10852.19 | 22.4/77.6 | NA |
|  | Cubic | 9282.45 | 9336.53 | 47.1/52.9 | NA |
| 3 | Linear | 11339.72 | 11388.88 | 7.5/77.7/14.8 | 0.82/0.91/0.99 |
|  | Quadratic | 10665.56 | 10734.39 | 19.1/35.2/45.7 | 0.97/0.99/1.00 |
|  | Cubic | 9183.35 | 9252.18 | 36.9/15.8/47.3 | 0.99/0.99/1.00 |
| 4 | Linear | 11189.28 | 11253.19 | 74.2/7.5/11.2/7.1 | 0.98/0.67/0.83/0.43 |
|  | Quadratic | 10370.46 | 10458.96 | 18.6/18.4/8.3/54.7 | 0.99/0.98/0.44/0.92 |
|  | Cubic | 9152.52 | 9236.1 | 28.4/29.7/26.5/15.4 | 0.98/0.99/0.62/1.00 |
| 5 | Linear | 11000.69 | 11099.03 | 2.6/7.2/10.2/6.8/73.2 | 0.97/0.37/0.99/0.57/0.98 |
|  | Quadratic | NA | NA | NA | NA |
|  | Cubic | NA | NA | NA | NA |
| 6 | Linear | 10773.40 | 10866.9 | 3.0/7.0/13.3/5.3/32.5/38.9 | 0.97/0.73/0.91/0.96/1.00/0.99 |
|  | Quadratic | 10270.52 | 10398.35 | 23.7/3.9/15.7/16.3/33.3/7.1 | 0.95/0.47/0.99/0.98/0.97/0.93 |
|  | Cubic | 8942.92 | 9056 | 12.8/25.3/6.2/13.6/25.3/16.8 | 0.98/0.99/0.72/1.00/0.91/0.81 |

**Table S3. Parameter estimates for the best fitting 3-class quadratic latent class growth mixture model fitted to the α-fetoprotein (AFP) data.**

| **Polynomial term** | **Class** | **Coefficient** | **Standard error** | **Wald** | **P-value** |
| --- | --- | --- | --- | --- | --- |
| **Linear** | class1-high-stable | -0.02223 | 0.00262 | -8.48000 | <0.001 |
|  | class2-sharp-falling | -0.56470 | 0.00862 | -65.50600 | <0.001 |
|  | class3-low-stable | -0.26203 | 0.00584 | -44.84700 | <0.001 |
| **Quadratic** | class1-high-stable | -0.05093 | 0.04279 | -1.19000 | <0.001 |
|  | class2-sharp-falling | -0.51981 | 0.03457 | -15.03700 | <0.001 |
|  | class3-low-stable | -0.27870 | 0.03078 | -9.05500 | <0.001 |
| **Cubic** | class1-high-stable | -0.42698 | 0.02160 | -19.76700 | <0.001 |
|  | class2-sharp-falling | -0.04416 | 0.00626 | -7.05400 | <0.001 |
|  | class3-low-stable | -0.03925 | 0.00764 | -5.14000 | <0.001 |

**Table S4.** Estimated slope and value of AFP in different points by 3-class quadratic LCGMM model.

| **Time (weeks)** | **Slope of high-stable** | **Slope of sharp-falling** | **Slope of low-stable** |
| --- | --- | --- | --- |
| 2 | -0.0136 | -0.1377 | -0.0246 |
| 4 | -0.0381 | -0.3012 | -0.0398 |
| 6 | -0.0651 | -0.2289 | -0.0344 |
| 8 | -0.0350 | -0.1970 | -0.0250 |
| 10 | -0.0500 | -0.1131 | 0.0000 |
| 12 | -0.0350 | -0.1312 | 0.0350 |
| 14 | -0.0150 | -0.1189 | 0.0150 |
| 16 | -0.0500 | -0.1497 | 0.0100 |
| 18 | -0.0700 | -0.1050 | 0.0300 |

**Table S5.** The coefficients of HCC patients among different groups after inverse-probability-of-treatment weighted analysis (IPTW)

| **Coefficients:** | **Estimate** | **Std. Error** | **z-value** | **Pr(>|z|)** |
| --- | --- | --- | --- | --- |
| (Intercept) | 0.90995 | 0.48126 | 1.891 | 0.0587 |
| **Age** | -0.04227 | 0.15706 | -0.269 | 0.7878 |
| **Etiology** | -0.16109 | 0.26092 | -0.617 | 0.5370 |
| **Comorbidity** | -0.02021 | 0.20195 | -0.100 | 0.9203 |
| **ALBI stage** | 0.06040 | 0.32685 | 0.185 | 0.8534 |
| **Tumor size** | -0.11213 | 0.15293 | -0.733 | 0.4634 |
| **Ascites** | 0.01409 | 0.19841 | 0.071 | 0.9434 |
| **Tumor number** | 0.04334 | 0.13942 | 0.311 | 0.7559 |
| **Vascular invasion** | 0.11677 | 0.24496 | 0.477 | 0.6336 |
| **BCLC stage** | -0.10655 | 0.18076 | -0.589 | 0.5556 |

**NOTE.** Null deviance: 1328.2 on 1008 degrees of freedom; Residual deviance: 1326.8 on 999 degrees of freedom; AIC: 1465.9. The patients in different groups were adjusted with age, etiology, comorbidity, ALBI stage, tumor size, Ascites, tumor number, vascular invasion and BCLC stage.

**Table S6.** Subgroup analysis of overall survival, progression-free survival and intrahepatic recurrence-free survival between High-stable and Sharp-falling groups by clinical features

| **Variables** | **OS** | **PFS** | **IRFS** |
| --- | --- | --- | --- |
|  | **HR (95CI) & P-value** | **HR (95CI) & P- value** | **HR (95CI) & P- value** |
| Age (≤65) | 0.21 (0.14-0.30, p<.001) | 0.27 (0.20-0.36, p<.001) | 0.23 (0.16-0.32, p<.001) |
| Age (>65) | 0.11 (0.05-0.22, p<.001) | 0.15 (0.08-0.28, p<.001) | 0.16 (0.08-0.30, p<.001) |
| Gender (Female) | 0.24 (0.10-0.57, p=.001) | 0.18 (0.07-0.43, p<.001) | 0.19 (0.07-0.52, p=.001) |
| Gender (Male) | 0.16 (0.12-0.23, p<.001) | 0.23 (0.18-0.31, p<.001) | 0.20 (0.14-0.28, p<.001) |
| ECOG-0 | 0.18 (0.13-0.25, p<.001) | 0.24 (0.18-0.31, p<.001) | 0.21 (0.15-0.28, p<.001) |
| ECOG-1 | 0.07 (0.01-0.60, p=.015) | 0.15 (0.03-0.67, p=.013) | 0.16 (0.03-0.71, p=.016) |
| Comorbidity (Absence) | 0.17 (0.12-0.25, p<.001) | 0.23 (0.17-0.30, p<.001) | 0.19 (0.14-0.27, p<.001) |
| Comorbidity (Presence) | 0.17 (0.06-0.47, p=.001) | 0.26 (0.12-0.55, p<.001) | 0.30 (0.13-0.66, p=.003) |
| Etiology (Other) | 0.10 (0.02-0.48, p=.004) | 0.16 (0.05-0.49, p=.001) | 0.18 (0.06-0.58, p=.004) |
| Etiology (HBV) | 0.18 (0.13-0.25, p<.001) | 0.24 (0.18-0.31, p<.001) | 0.20 (0.15-0.28, p<.001) |
| Ascites (Absence) | 0.18 (0.12-0.25, p<.001) | 0.25 (0.19-0.33, p<.001) | 0.22 (0.16-0.31, p<.001) |
| Ascites (Presence) | 0.19 (0.07-0.48, p<.001) | 0.12 (0.05-0.32, p<.001) | 0.09 (0.03-0.29, p<.001) |
| PALBI grade (1) | 0.19 (0.13-0.29, p<.001) | 0.20 (0.14-0.29, p<.001) | 0.18 (0.12-0.27, p<.001) |
| PALBI grade (2&3) | 0.15 (0.09-0.26, p<.001) | 0.26 (0.18-0.40, p<.001) | 0.24 (0.15-0.38, p<.001) |
| Tumor size (5-7] | 0.18 (0.09-0.35, p<.001) | 0.15 (0.08-0.29, p<.001) | 0.11 (0.05-0.24, p<.001) |
| Tumor size (>7) | 0.18 (0.12-0.25, p<.001) | 0.26 (0.20-0.35, p<.001) | 0.24 (0.17-0.34, p<.001) |
| Tumor number (≤3) | 0.13 (0.08-0.22, p<.001) | 0.19 (0.12-0.29, p<.001) | 0.18 (0.11-0.30, p<.001) |
| Tumor number (>3) | 0.23 (0.15-0.35, p<.001) | 0.30 (0.21-0.42, p<.001) | 0.24 (0.16-0.36, p<.001) |
| Vascular invasion (Absence) | 0.14 (0.07-0.28, p<.001) | 0.24 (0.14-0.39, p<.001) | 0.24 (0.14-0.41, p<.001) |
| Vascular invasion (Presence) | 0.19 (0.13-0.28, p<.001) | 0.24 (0.17-0.32, p<.001) | 0.20 (0.13-0.29, p<.001) |
| Metastasis (Absence) | 0.16 (0.11-0.24, p<.001) | 0.21 (0.15-0.30, p<.001) | 0.17 (0.11-0.25, p<.001) |
| Metastasis (Presence) | 0.22 (0.13-0.37, p<.001) | 0.29 (0.19-0.44, p<.001) | 0.30 (0.19-0.48, p<.001) |
| BCLC-A | 0.14 (0.04-0.46, p=.001) | 0.30 (0.12-0.74, p=.009) | 0.40 (0.16-1.03, p=.057) |
| BCLC-B | 0.15 (0.06-0.41, p<.001) | 0.24 (0.12-0.51, p<.001) | 0.21 (0.09-0.47, p<.001) |
| BCLC-C | 0.19 (0.13-0.28, p<.001) | 0.23 (0.17-0.32, p<.001) | 0.19 (0.13-0.28, p<.001) |

**NOTE:** The subgroup analysis was developed with Cox regression analyses based on overall survival (OS), progression-free survival (PFS) and intrahepatic recurrence-free survival (IRFS).

**Abbreviations:** HCC, hepatocellular carcinoma; HAIC, hepatic arterial infusion chemotherapy; HRs, hazard ratios; CI, confidence interval; ALBI, Albumin-Bilirubin; OS: Overall survival; PFS: Progression-free survival; IRFS: Intrahepatic recurrence-free survival.

**Table S7.** Subgroup analysis of overall survival, progression-free survival and intrahepatic recurrence-free survival between High-stable and Low-stable groups by clinical features

| **Variables** | **OS** | **PFS** | **IRFS** |
| --- | --- | --- | --- |
|  | **HR (95CI) & P-value** | **HR (95CI) & P- value** | **HR (95CI) & P- value** |
| Age (≤65) | 0.50 (0.41-0.61, p<.001) | 0.62 (0.52-0.74, p<.001) | 0.60 (0.49-0.73, p<.001) |
| Age (>65) | 0.49 (0.34-0.72, p<.001) | 0.49 (0.34-0.69, p<.001) | 0.49 (0.33-0.71, p<.001) |
| Gender (Female) | 0.77 (0.47-1.25, p=.287) | 0.70 (0.43-1.14, p=.151) | 0.73 (0.42-1.27, p=.263) |
| Gender (Male) | 0.46 (0.38-0.56, p<.001) | 0.56 (0.47-0.66, p<.001) | 0.55 (0.46-0.65, p<.001) |
| ECOG-0 | 0.48 (0.41-0.58, p<.001) | 0.58 (0.49-0.68, p<.001) | 0.58 (0.48-0.69, p<.001) |
| ECOG-1 | 0.87 (0.38-1.97, p=.736) | 0.49 (0.23-1.04, p=.063) | 0.37 (0.16-0.86, p=.022) |
| Comorbidity (Absence) | 0.51 (0.42-0.61, p<.001) | 0.60 (0.51-0.71, p<.001) | 0.60 (0.50-0.71, p<.001) |
| Comorbidity (Presence) | 0.51 (0.30-0.88, p=.015) | 0.44 (0.27-0.71, p=.001) | 0.43 (0.26-0.73, p=.002) |
| Etiology (Other) | 0.51 (0.25-1.01, p=.054) | 0.66 (0.36-1.22, p=.187) | 0.65 (0.33-1.29, p=.222) |
| Etiology (HBV) | 0.50 (0.41-0.59, p<.001) | 0.57 (0.49-0.67, p<.001) | 0.57 (0.47-0.68, p<.001) |
| Ascites (Absence) | 0.50 (0.42-0.60, p<.001) | 0.57 (0.49-0.68, p<.001) | 0.57 (0.47-0.68, p<.001) |
| Ascites (Presence) | 0.51 (0.31-0.83, p=.007) | 0.70 (0.46-1.07, p=.102) | 0.64 (0.39-1.05, p=.079) |
| PALBI grade (1) | 0.71 (0.63-0.80, p<.001) | 0.70 (0.63-0.78, p<.001) | 0.71 (0.63-0.80, p<.001) |
| PALBI grade (2&3) | 0.70 (0.62-0.80, p<.001) | 0.82 (0.73-0.91, p<.001) | 0.80 (0.71-0.90, p<.001) |
| Tumor size (5-7] | 0.59 (0.41-0.85, p=.004) | 0.62 (0.45-0.86, p=.004) | 0.61 (0.43-0.87, p=.007) |
| Tumor size (>7) | 0.48 (0.39-0.58, p<.001) | 0.57 (0.48-0.68, p<.001) | 0.56 (0.46-0.68, p<.001) |
| Tumor number (≤3) | 0.50 (0.38-0.67, p<.001) | 0.61 (0.47-0.79, p<.001) | 0.61 (0.46-0.82, p=.001) |
| Tumor number (>3) | 0.51 (0.41-0.64, p<.001) | 0.58 (0.48-0.70, p<.001) | 0.56 (0.45-0.69, p<.001) |
| Vascular invasion (Absence) | 0.51 (0.36-0.73, p<.001) | 0.55 (0.40-0.76, p<.001) | 0.55 (0.39-0.78, p=.001) |
| Vascular invasion (Presence) | 0.53 (0.43-0.64, p<.001) | 0.61 (0.51-0.72, p<.001) | 0.59 (0.49-0.72, p<.001) |
| Metastasis (Absence) | 0.44 (0.35-0.55, p<.001) | 0.60 (0.49-0.73, p<.001) | 0.58 (0.47-0.72, p<.001) |
| Metastasis (Presence) | 0.62 (0.48-0.80, p<.001) | 0.57 (0.45-0.73, p<.001) | 0.57 (0.43-0.75, p<.001) |
| BCLC-A | 0.52 (0.24-1.10, p=.088) | 0.62 (0.31-1.23, p=.171) | 0.68 (0.31-1.46, p=.319) |
| BCLC-B | 0.45 (0.27-0.73, p=.001) | 0.56 (0.36-0.85, p=.007) | 0.54 (0.34-0.86, p=.009) |
| BCLC-C | 0.54 (0.45-0.65, p<.001) | 0.59 (0.50-0.70, p<.001) | 0.57 (0.47-0.70, p<.001) |

**NOTE:** The subgroup analysis was developed with Cox regression analyses based on overall survival (OS), progression-free survival (PFS) and intrahepatic recurrence-free survival (IRFS).

**Abbreviations:** HCC, hepatocellular carcinoma; HAIC, hepatic arterial infusion chemotherapy; HRs, hazard ratios; CI, confidence interval; ALBI, Albumin-Bilirubin; OS: Overall survival; PFS: Progression-free survival; IRFS: Intrahepatic recurrence-free survival.

**Table S8.** Subgroup analysis of overall survival, progression-free survival and intrahepatic recurrence-free survival between Low-stable and Low-stable groups by clinical features

**NOTE:** The subgroup analysis was developed with Cox regression analyses based on overall survival (OS), progression-free survival (PFS) and intrahepatic recurrence-free survival (IRFS).

| **Variables** | **OS** | **PFS** | **IRFS** |
| --- | --- | --- | --- |
|  | **HR (95CI) & P-value** | **HR (95CI) & P- value** | **HR (95CI) & P- value** |
| Age (≤65) | 2.49 (1.72-3.60, p<.001) | 2.30 (1.71-3.10, p<.001) | 2.63 (1.85-3.75, p<.001) |
| Age (>65) | 4.05 (2.15-7.63, p<.001) | 3.20 (1.92-5.33, p<.001) | 3.34 (1.92-5.79, p<.001) |
| Gender (Female) | 3.43 (1.42-8.29, p=.006) | 4.07 (1.76-9.42, p=.001) | 3.93 (1.58-9.76, p=.003) |
| Gender (Male) | 2.78 (1.98-3.92, p<.001) | 2.41 (1.84-3.16, p<.001) | 2.74 (2.00-3.76, p<.001) |
| ECOG-0 | 2.78 (2.01-3.85, p<.001) | 2.49 (1.92-3.23, p<.001) | 2.84 (2.09-3.84, p<.001) |
| ECOG-1 | 4.86 (1.11-21.36, p=.036) | 3.85 (0.87-17.06, p=.076) | 2.86 (0.62-13.17, p=.176) |
| Comorbidity (Absence) | 2.98 (2.12-4.20, p<.001) | 2.73 (2.06-3.61, p<.001) | 3.12 (2.25-4.33, p<.001) |
| Comorbidity (Presence) | 2.32 (0.98-5.51, p=.056) | 1.82 (0.94-3.52, p=.074) | 1.74 (0.84-3.60, p=.135) |
| Etiology (Other) | 4.59 (1.35-15.62, p=.015) | 5.52 (1.93-15.84, p=.001) | 4.59 (1.57-13.39, p=.005) |
| Etiology (HBV) | 2.76 (1.98-3.84, p<.001) | 2.40 (1.84-3.13, p<.001) | 2.75 (2.02-3.76, p<.001) |
| Ascites (Absence) | 2.87 (2.05-4.02, p<.001) | 2.35 (1.80-3.07, p<.001) | 2.64 (1.94-3.59, p<.001) |
| Ascites (Presence) | 2.67 (1.02-7.00, p=.045) | 5.55 (2.15-14.32, p<.001) | 6.80 (2.03-22.82, p=.002) |
| PALBI grade (1) | 2.39 (1.61-3.56, p<.001) | 2.41 (1.72-3.38, p<.001) | 2.84 (1.90-4.24, p<.001) |
| PALBI grade (2&3) | 3.70 (2.16-6.35, p<.001) | 2.65 (1.78-3.94, p<.001) | 2.76 (1.77-4.30, p<.001) |
| Tumor size (5-7] | 3.46 (1.78-6.73, p<.001) | 4.11 (2.25-7.50, p<.001) | 5.66 (2.60-12.29, p<.001) |
| Tumor size (>7) | 2.68 (1.87-3.86, p<.001) | 2.23 (1.68-2.98, p<.001) | 2.42 (1.74-3.35, p<.001) |
| Tumor number (≤3) | 3.51 (2.10-5.87, p<.001) | 3.11 (2.06-4.68, p<.001) | 3.22 (2.03-5.10, p<.001) |
| Tumor number (>3) | 2.34 (1.56-3.52, p<.001) | 2.07 (1.48-2.88, p<.001) | 2.46 (1.66-3.65, p<.001) |
| Vascular invasion (Absence) | 3.15 (1.72-5.78, p<.001) | 2.50 (1.58-3.96, p<.001) | 2.50 (1.51-4.15, p<.001) |
| Vascular invasion (Presence) | 2.78 (1.91-4.04, p<.001) | 2.57 (1.89-3.51, p<.001) | 3.04 (2.10-4.40, p<.001) |
| Metastasis (Absence) | 2.70 (1.81-4.04, p<.001) | 2.75 (1.99-3.81, p<.001) | 3.39 (2.30-5.00, p<.001) |
| Metastasis (Presence) | 2.92 (1.72-4.95, p<.001) | 2.11 (1.39-3.21, p<.001) | 2.07 (1.30-3.30, p=.002) |
| BCLC-A | 2.37 (0.88-6.42, p=.088) | 2.10 (0.98-4.48, p=.055) | 2.10 (0.98-4.48, p=.055) |
| BCLC-B | 3.02 (1.19-7.67, p=.020) | 2.18 (1.11-4.26, p=.023) | 2.36 (1.12-4.98, p=.024) |
| BCLC-C | 2.86 (1.99-4.10, p<.001) | 2.56 (1.89-3.45, p<.001) | 2.98 (2.09-4.27, p<.001) |

**Abbreviations:** HCC, hepatocellular carcinoma; HAIC, hepatic arterial infusion chemotherapy; HRs, hazard ratios; CI, confidence interval; ALBI, Albumin-Bilirubin; OS: Overall survival; PFS: Progression-free survival; IRFS: Intrahepatic recurrence-free survival.

1.
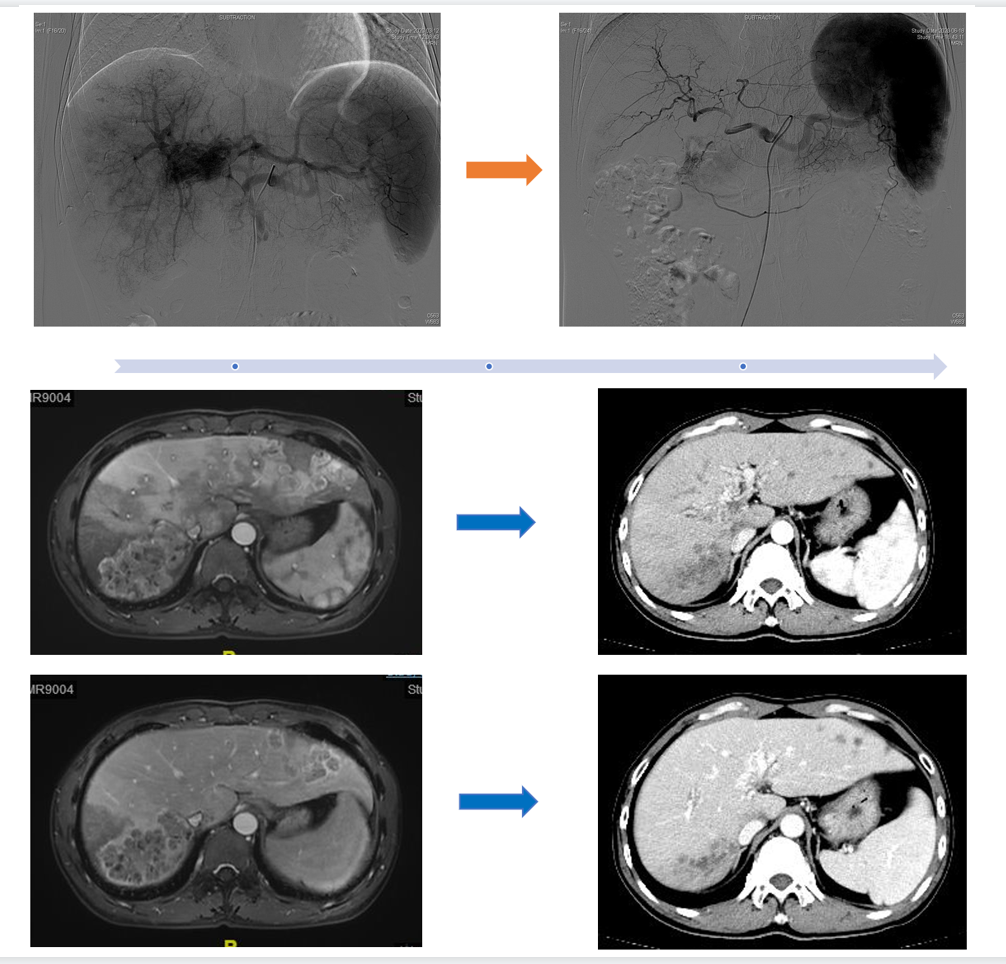
**Supplementary Figures**

**Figure. S1** An example of patients with large HCC receiving HAIC plus lenvatinib. After four cycles of HAIC, the large HCC shrank significantly (the tumor diameter from 12.8 cm to 7.5 cm), and then one month after HAIC treatment, the tumor thrombus disappeared with oral lenvatinib.


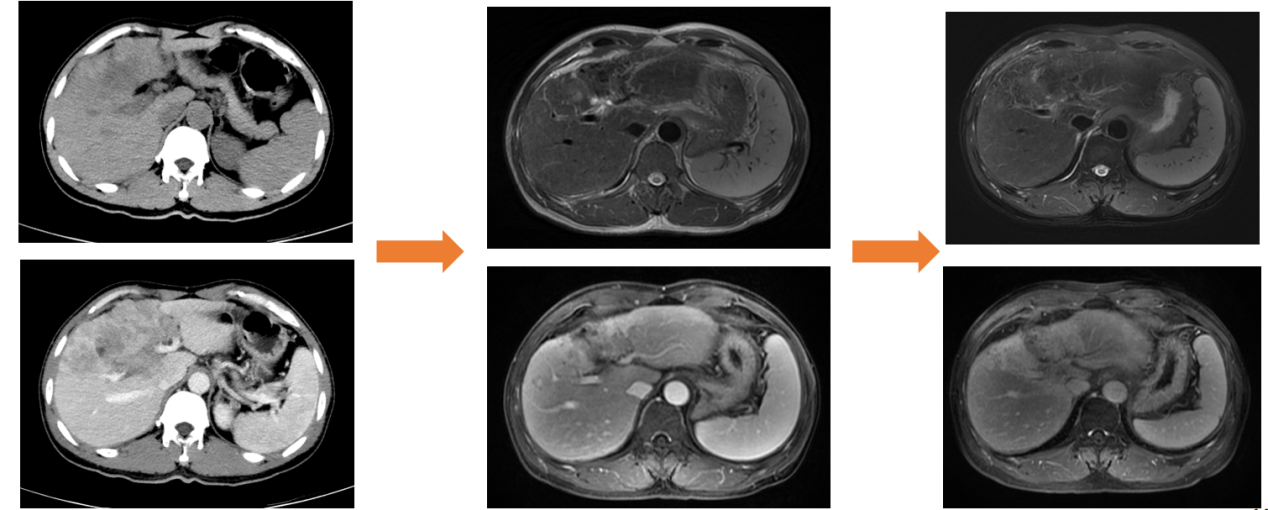
**Figure. S2** An example of patients with large HCC receiving HAIC plus PD-1. After six cycles of HAIC, the large HCC shrank significantly (the tumor diameter from 11.4 cm to 4.8 cm), and then one day after HAIC treatment, the tumor thrombus disappeared when PD-1 was infused intravenously.


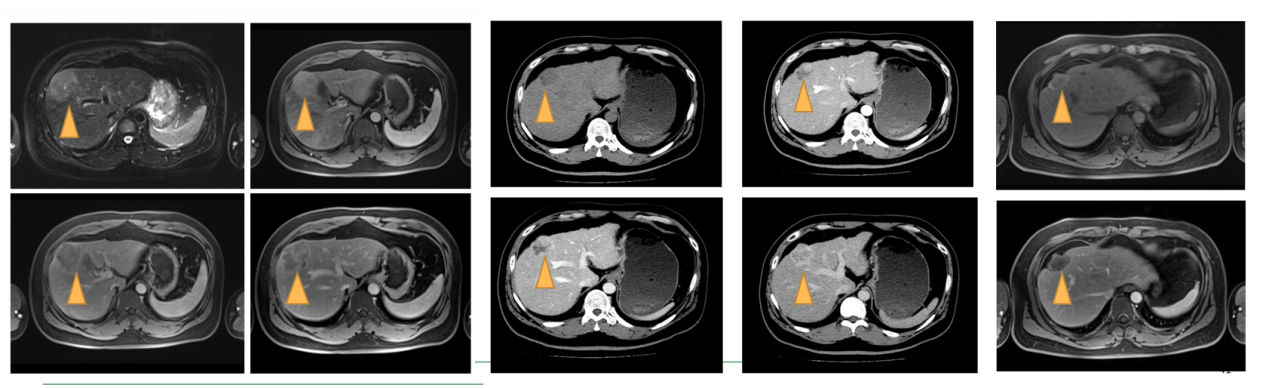

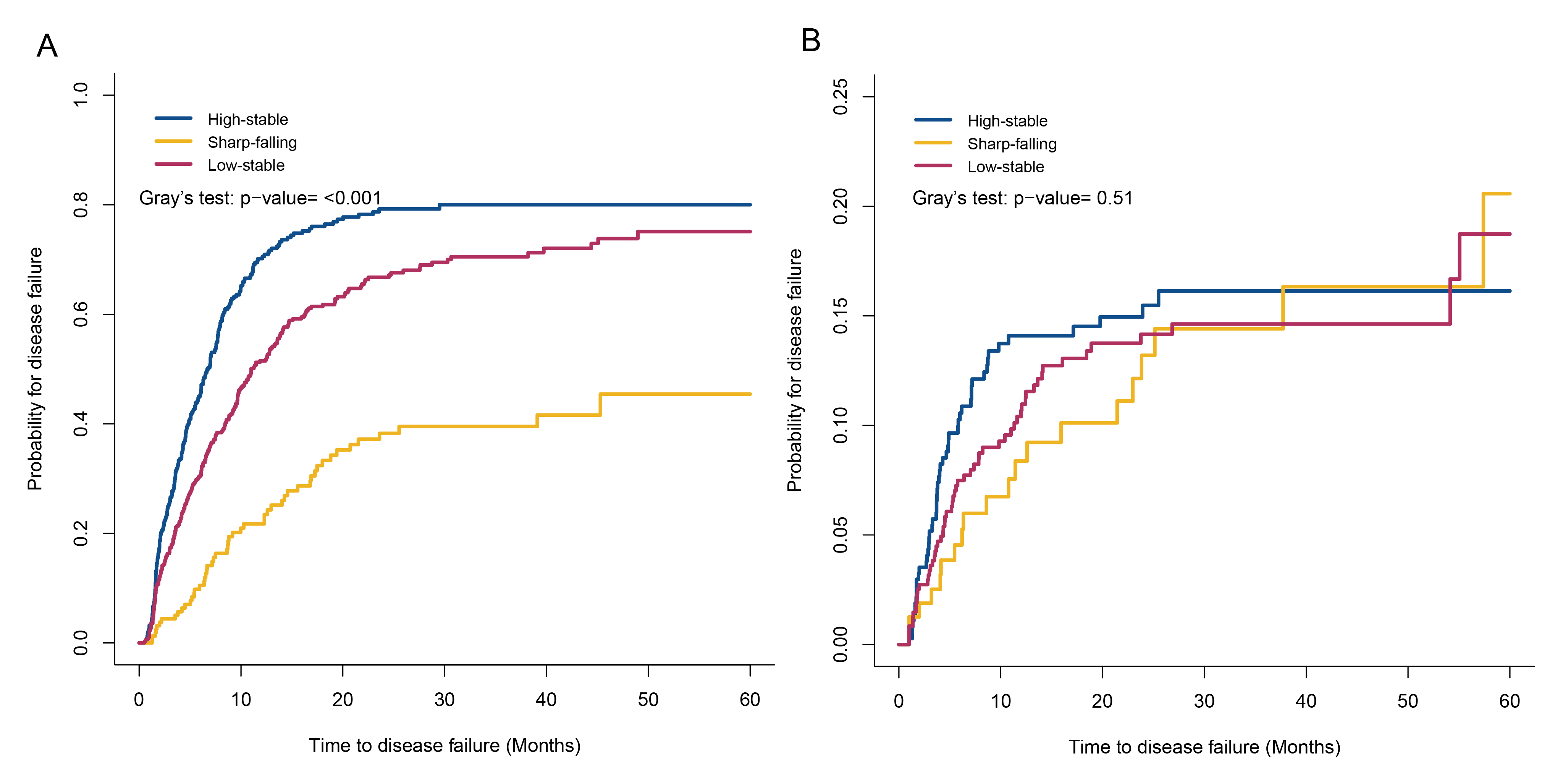
 **Figure. S3** An example of patients with large HCC receiving HAIC combined with sequential RFA. After four cycles of HAIC, the large HCC shrank significantly (the tumor diameter from 5.2 cm to 2.3cm, nodule with yellow arrow), and then 42 days after HAIC treatment RFA was used, eradicating the large HCC with complete ablation.

**Figure. S4** Comparisons of cumulative incidence of intrahepatic recurrence-free survival (IRFS) among patients in different AFP trajectories groups. (A) for IRFS; (B) for a non- IRFS or all-cause death were considered competing events. P values were calculated with the Gray test

**Abbreviations:** AFP, a-fetoprotein; BCLC, Barcelona Clinic Liver Cancer; ALBI, Albumin Bilirubin; HCC, hepatocellular carcinoma; HAIC, hepatic arterial infusion chemotherapy; IRFS: Intrahepatic recurrence-free survival; PFS: Progression-free survival; OS: Overall survival.


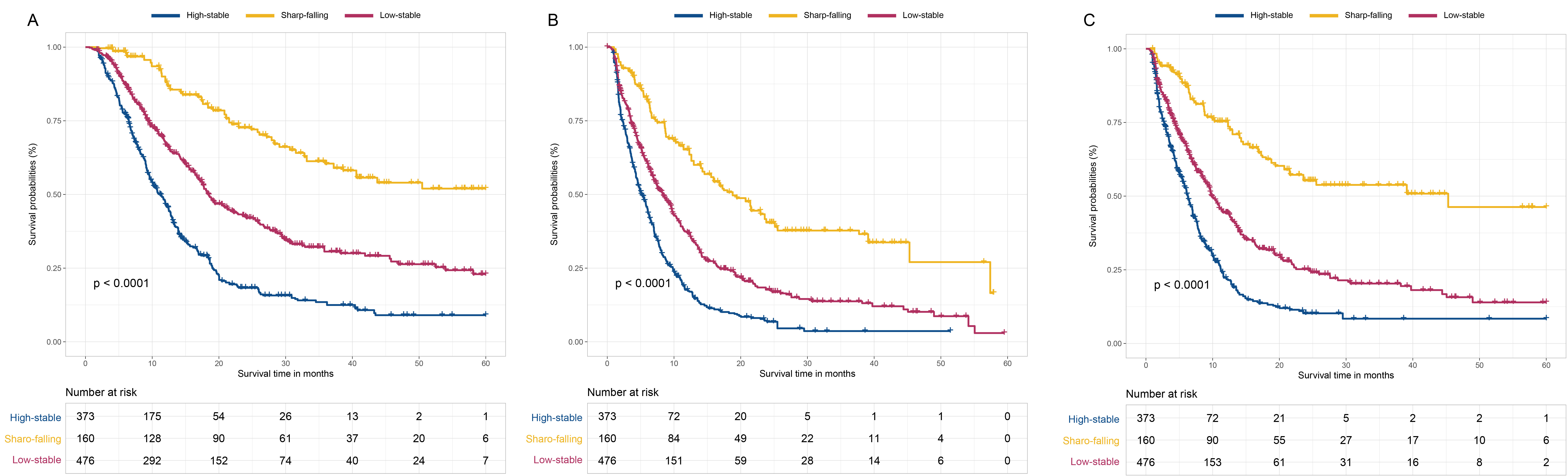


**Figure. S5** The inverse-probability-of-treatment weighted analysis (IPTW) for HCC patients after HAIC in different BCLC stages. Comparing the overall survival (OS)(A), progression-free survival (PFS) (B) and intrahepatic recurrence-free survival (IRFS) (C) among different AFP Trajectories groups of BCLC A stage. The patients in different groups were adjusted with age, etiology, comorbidity, ALBI stage, tumor size, Ascites, tumor number, vascular invasion and BCLC stage.

**Abbreviations:** AFP, a-fetoprotein; BCLC, Barcelona Clinic Liver Cancer; ALBI, Albumin Bilirubin; HCC, hepatocellular carcinoma; HAIC, hepatic arterial infusion chemotherapy; IRFS: Intrahepatic recurrence-free survival; PFS: Progression-free survival; OS: Overall survival.


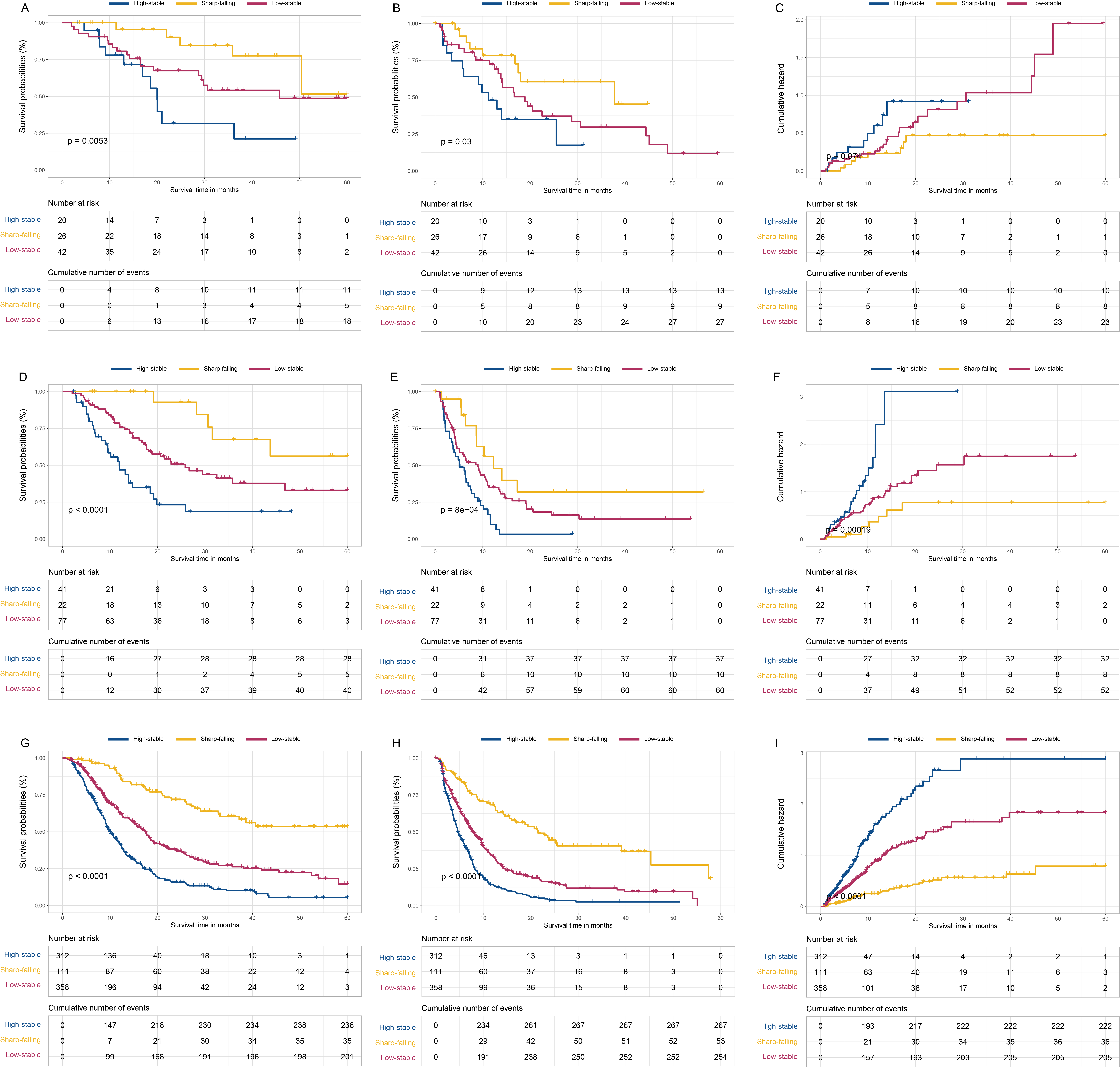
**Figure. S6** Subgroup analysis of HCC patients after HAIC in different BCLC stages. Comparing the overall survival (OS)(A), progression-free survival (PFS) (B) and intrahepatic recurrence-free survival (IRFS) (C) among different AFP trajectories groups of BCLC A stage. Comparing the OS (D), PFS (E) and IRFS (F) among different AFP Trajectories groups of BCLC B stage. Comparing the OS (G), PFS (H) and IRFS (I) among different AFP Trajectories groups of BCLC C stage.

**Abbreviations:** AFP, a-fetoprotein; BCLC, Barcelona Clinic Liver Cancer; ALBI, Albumin Bilirubin; HCC, hepatocellular carcinoma; HAIC, hepatic arterial infusion chemotherapy; IRFS: Intrahepatic recurrence-free survival; PFS: Progression-free survival; OS: Overall survival.
